# Neuropeptide S modulation of learning and memory: a systematic review

**DOI:** 10.1186/s12993-026-00336-y

**Published:** 2026-05-03

**Authors:** Violeta-Maria Caragea, Kay Jüngling, Marta Méndez-Couz

**Affiliations:** 1 Research Institute of the University of Bucharest, Șoseaua Panduri, 90-92, 5th District, Bucharest, 050663 Romania; 2 Department of Anatomy, Animal Physiology and Biophysics, Faculty of Biology, University of Bucharest, Splaiul Independenței 91-95, Bucharest, 050095 Romania; 3 Institute of Physiology I, Münster University, Robert-Koch-Str. 27A, 48149 Münster, Germany

**Keywords:** NPSR1, Neuromodulation, Neuropeptides, Long-term memory, Fear memory, Reward, Extinction, Addiction, Anxiety

## Abstract

**Background:**

In the last two decades, Neuropeptide S (NPS) has been identified as a key bioactive peptide in the mammalian brain, influencing fear, anxiety, wakefulness, reward, and learning. While some reviews have addressed its role in reward-seeking and anxiety, few have addressed its particular role in learning and memory. The neuropeptide S receptor 1 is highly expressed in key areas for learning processing, such as the hippocampus, cortex, thalamus, and amygdala. This review aims to examine evidence from human and animal studies that focused on the NPS system’s role in modulating learning and memory. A special focus is given to experiments addressing the impact of NPS on associative learning leading to addiction and in fear conditioning, pointing to its potential therapeutic value in associated pathologies.

**Main body:**

An advanced search was conducted using the databases PubMed, Google Scholar, Web of Science, and Scopus, focusing on memory and Neuropeptide S. The reviewed data suggest that NPS modulation occurs at all memory phases, including acquisition, consolidation, and retrieval, and in extinction learning, whether motivated by appetitive or aversive stimuli. The summarized evidence shows that the NPS system interferes with working and short-term memory, mitigates learning impairments, enhances spatial and object memory consolidation, supports fear extinction learning and inhibitory avoidance consolidation, and reinstates drug-seeking behaviors. The NPS system closely interacts with key neuromodulators, including orexinergic, dopaminergic, and noradrenergic systems, in influencing memory.

**Conclusion:**

The Neuropeptide S system emerges as a critical modulator of memory processes. The NPS signaling may preferentially influence learning that involves emotionally or motivationally relevant stimuli. This highlights the NPS system’s potential as a target for therapeutic interventions for particular memory impairments.

**Supplementary Information:**

The online version contains supplementary material available at 10.1186/s12993-026-00336-y.

## Introduction

Neuropeptide S (NPS) is a small neuropeptide of only 20 amino acids, highly conserved in tetrapod vertebrates, including rodents and humans [1]. NPS-producing neurons are located in a limited number of brain structures, similar in rodents and humans [2, 3]. Specifically, in rats, the NPS precursor mRNA has its highest expression in the brainstem, particularly in the peri-coerulean region (periLC), the principle 5 sensory nucleus neurons, which is void of NPS mRNA in mice, and the lateral parabrachial nucleus [4]. A significantly lower expression was found in the amygdaloid complex and several hypothalamic nuclei [4]. Additionally, in the mouse brain, it was located in the periLC, between the lateral parabrachial nucleus and the Kölliker-Fuse nucleus, and the anterior olfactory nucleus [3, 5, 6]. NPS-expressing neurons were also identified in mice in the nucleus incertus and the anterior hypothalamus [7].

NPS binds to a G-protein-coupled receptor, the neuropeptide S receptor (NPSR, or NPSR1) [4]. In contrast to NPS, the receptor is widely expressed in the brain, with relatively high levels in the hippocampal formation, cortex, thalamus, hypothalamus, olfactory nuclei and amygdala, as well as in lower levels in the brainstem [3, 8]. A strong overlap of the *Npsr1* mRNA expression and NPS-immunoreactivity was found in hypothalamic areas such as the paraventricular nucleus (PVN) [9]. In rats, *Npsr1* mRNA is strongly expressed in the amygdala, hypothalamus, secondary motor cortex, and retrosplenial agranular cortex [4]. In mice, NPSR1 expression was found in the basolateral nucleus of the amygdala (BLA) and the endopiriform cortex [10]. Activation of NPSR1 leads to an increase of intracellular Ca^2+^, cAMP and MAPK activation upon stimulation by NPS, thus indicating an excitatory effect of NPS [11–14].

Functional studies where *Npsr1* gene or NPSR1 protein were either genetically or pharmacologically manipulated reported that the NPS system plays important regulatory roles in fear and anxiety [15], arousal [1], wakefulness [16], sleep architecture [17], and reward-seeking and addiction [18]. Although multiple studies reported on NPS system’s effects on various forms of learning and memory (i.e. [19–23]), no review focusing on this alone has yet been published.

The association of the NPS/NPSR1 system with stress-related disorders has been noted through genetic variants in the* NPSR1* gene (rs324981: A/T mutation at chr7:34778501 [GRCh38.p14]) [12, 24]. The A allele, which encodes the *NPSR1*-Asn107 variant (N107), exhibits lower potency than the T-encoded *NPSR1*Ile107 variant (I107) [12, 25]. Although for years, the ancestral T allele was thought to confer a gain of function of the human receptor vs. the A allele [12, 25], recent genetic studies suggest that the A polymorphism, found exclusively in modern humans, promotes a reduced functionality of the transmitter system [26]. These authors suggest that the hypofunctional A allele could have evolved in modern humans to enhance prosocial behaviors and adaptability [26].

This systematic review focuses on preclinical research that reveals a role for NPS in long and short-term forms of memory. This includes the most used forms of fear conditioning: classical or instrumental, positive reinforcement for operant conditioning, and other forms of learning driven by appetitive or neutral stimuli, such as spatial learning, sensory memory or object recognition, and social memory. Additionally, we review here the implications of the NPS system for the mechanisms and circuitries of memory acquisition, consolidation, retrieval, and the extinction learning and memory process. In the case of fear memory and addiction models, most of the studies reviewed focused on the mechanisms of extinction learning and its relapse (reinstatement or renewal), given its therapeutic importance. Since NPS has an anxiolytic effect [4], we included the impact of NPS on anxiety-related behaviors, often analyzed in studies focusing on memory, given its potential interfering effects. Finally, we will discuss some of the modulatory systems interacting with the NPS system in memory.

Importantly, the diversity of learning paradigms influenced by the NPS system raises the question of whether its primary role is mnemonic in nature or whether it acts as a modulator of the underlying factors important for memory functioning. Across the reviewed studies, NPS effects are most consistently observed when learned associations compete for behavioral expression, such as during extinction learning [27], reinstatement [28], and generalization [29]. This suggests that the NPS effects appear most prominent in learning situations involving emotionally or motivationally relevant stimuli, or requiring cognitive flexibility, further supporting an indirect modulatory role of the NPS system, rather than a uniform enhancement of memory acquisition and storage.

With this review, we seek to contribute to both understanding the NPS system role in memory from molecular, regional, and network perspective, and supporting the development and refinement of pharmacological interventions in cases of aberrant forms of learning, such as panic disorder and addictions.

## Methodology

### Search strategy

**Fig. 1:**
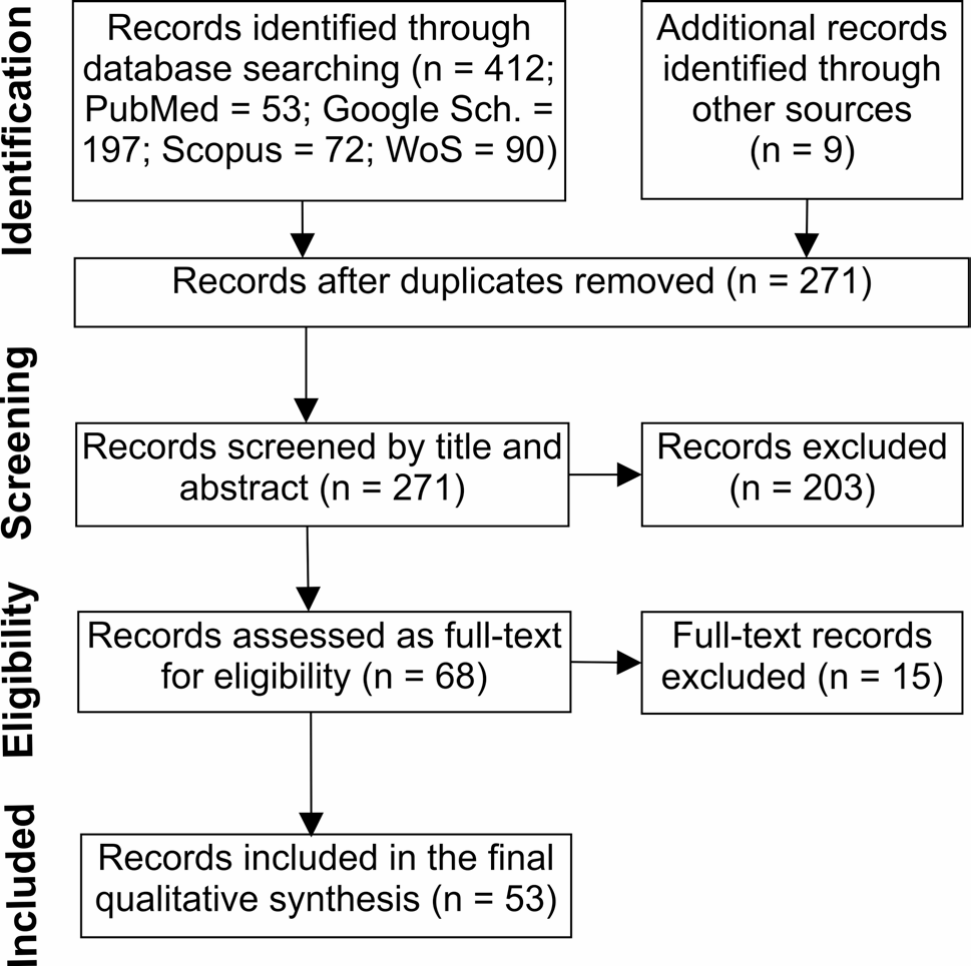
Search phases of the systematic review process

The review was conducted according to the Preferred Reporting Items for Systematic Reviews and Meta-Analyses (PRISMA) guidelines [30], with the search phases described in Fig. 1. The review protocol was not pre-registered before data collection. The database list and the inclusion and exclusion keywords were pre-selected in agreement with all co-authors, whereas the search was performed by a single author.

The search of relevant databases is updated to the 21st of August 2025 date, with no filters for the type of publication, language, or publication date. Four databases were queried: the PubMed database of the United States National Library of Medicine (“PubMed”), the Google Scholar bibliographic database (“Google Sch.”), the Web of Science platform of academic databases hosted by Clarivate (“WoS”), and the Scopus citation and abstract database by Elsevier (“Scopus”). This initial search led to a total of 412 records. Here, the strategy was customized for each database to filter the search results by the predefined keywords list (Supplementary Table S1). After screening these records, nine more research articles were identified and added to the list.

### Eligibility criteria and screening

After excluding 150 duplicates, an initial filtering of the resulting list of records was performed based on the following criteria, applied to the title and the abstract of each entry: (1) publications written in the English language; (2) publications that report original research findings; and (3) studies that focused on the role of the NPS system in learning and memory in non-neurodegenerative conditions. For the screening following the first two criteria, one researcher was involved, while for the third criterion, two researchers performed the screening process together and decided on the inclusion of each record based on the title and abstract. We decided to exclude studies using models of disorders that normally interfere with learning and memory (i.e., Alzheimer’s disease), but include the studies where aberrant learning leads to the installment of clinical manifestations (i.e., addiction). This screening step led to the exclusion of 203 records. We excluded: (1) studies with full-text versions published in a non-English language (23 records); (2) reviews, meta-analyses, theses, case-report studies, patents, editorials, letters, or conference abstracts and posters (51 records); (3) studies reporting data on the NPS system not directly approaching learning and memory (with direct behavioral evidence), or approaching this in clinical conditions not induced by aberrant learning (129 records).

The remaining 68 studies were divided between the two initial researchers, using an odd-even strategy, and their full text was thoroughly analyzed to decide on the inclusion. Apart from the criteria mentioned above, and to mitigate the potential risks of bias in the selected studies, the two reviewers assessed each included record for: 1) a clear description of the aim, research design, sample (including characterization of the model used), results, and conclusions of the study; 2) a reliable methodology (with controlling for potential confounding factors); and 3) a well-justified interpretation of the results. This further analysis led to the exclusion of 15 more records, based on lacking a clear focus on learning and memory, or focusing on neurodegenerative clinical factors in relation to NPS system and memory (i.e., by using drugs that induce neurodegeneration). In total, 53 records were included.

### Data synthesis and qualitative analysis strategy

Table 1 identifies the animal model and memory type investigated, and summarizes the key findings for each of the 53 selected studies of this review. A brief definition of the main memory-related terms used is provided in Table S2, while a more detailed information on population and animal models, sample distribution, NPS system and memory-related measurements, methodology, and results was included in the Supplementary Table 3 and 4, as follows: one sheet of data for human studies (Table S3), and two others including animal studies (rodents), the first referring to genetic interventions (Table S4a), and the second to pharmacological manipulations (Table S4b). The characteristics of the studies, their methodology and results are further discussed.

## Results

### Characteristics of the selected studies

From the 53 selected studies, only three investigated the NPS system in human memory (Table S3), while 50 tested its contribution to the memory of rodents. Within the latter group, 16 studies used genetic manipulations (Table S4a), whereas 39 articles reported results obtained from pharmacological interventions (Table S4b).

Mouse represented the most used animal model (36 studies), followed by rat (16 articles). All studies used adult human subjects or rodents. From the total of 53 studies, 15 used subjects or animals of both sexes: 35 used only males, one used only females, while two did not specify the sex of the animals. Only six studies using animals of both sexes in their tests regarding NPS(R1) and memory, reported sex-specific differences, while a seventh, including only female animals, reported differences in NPSR1-mediated behaviors as a factor of estrous cycle [31].

**Fig. 2:**
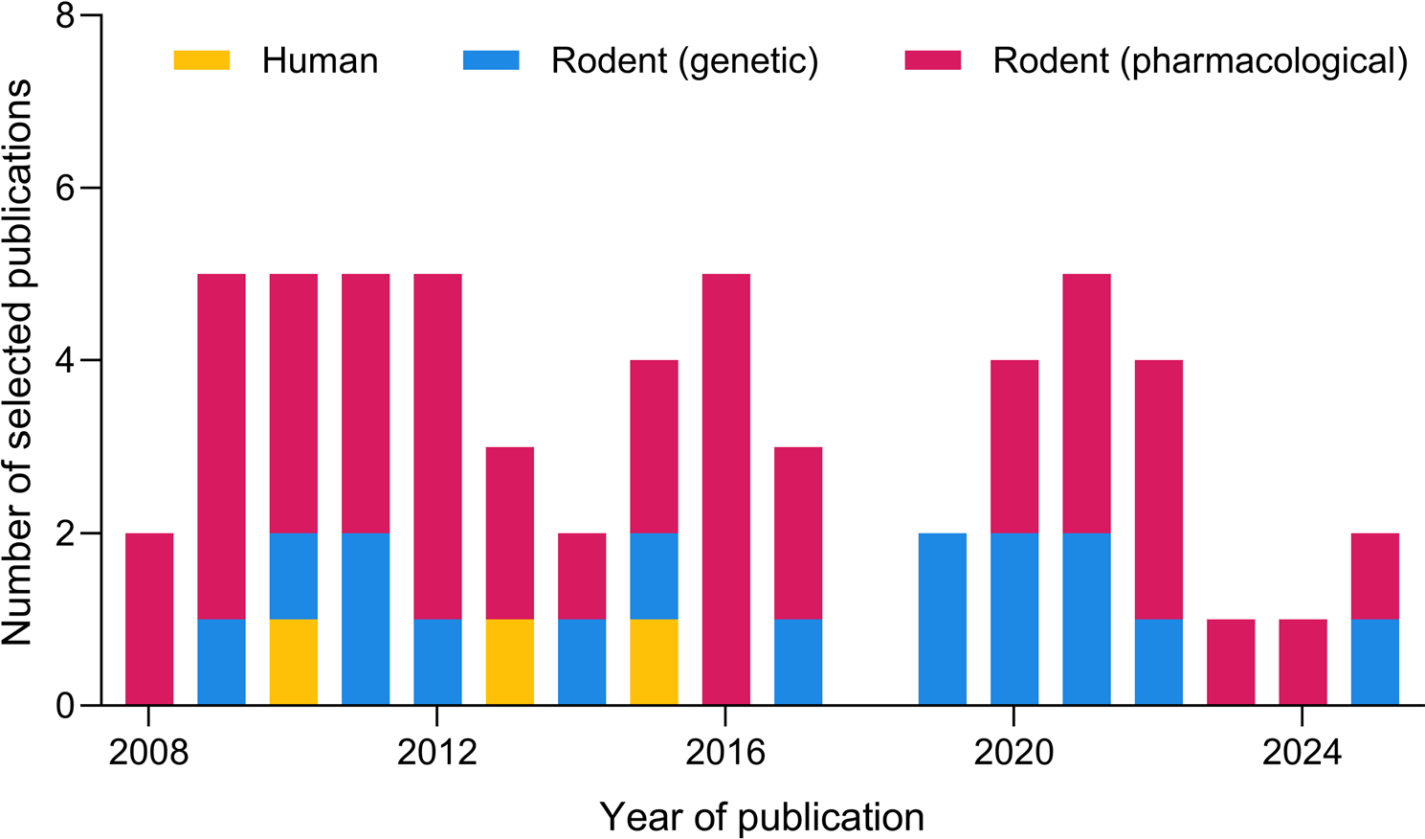
Temporal distribution of the selected studies

Various types of memory were addressed to investigate the modulatory effects of the NPS system on mnemonic processes, listed below in the order of their frequency:

1. Associative memory in the form of conditioning (either aversive - contextual, social, sensory-cued fear, learned helplessness; or appetitive - operant, drug-, alcohol-, or food-seeking), place preference or avoidance, inhibitory avoidance, and safety learning;
2. Episodic memory tested as the recognition of a novel object (or conspecific), place, or context;
3. Spatial memory, either short-term in the alternating T-maze task, or long-term in the water maze, and, in two cases, also combined with olfactory memory;
4. Working memory, measured with the n-back test, the reversal paradigm of the T-maze task, and the attentional set shifting task;
5. Motor learning, tested in the rotarod test.

Four papers addressing the role of the NPS system in the prepulse inhibition of the startle response, a form of sensorimotor gating [32], were also included in our selection due to its associative nature and relevance for working memory [33].

The NPS system role in memory was mostly scrutinized by looking at behavioral effects of pharmacological ligands targeting NPSR1, or by genetically engineering these receptors. Several papers also employed immunohistochemistry methods to analyze neuronal markers such as c-Fos immunoreactivity after NPS administration or during the activity and connectivity of NPS-expressing neurons related to memory-relevant brain regions.

The main effects related to the NPS system and memory identified in the selected pool of studies are described in the next section (3.2 *Qualitative synthesis of the included studies*). For more detailed information on the methodology and specific results, refer to the Table S3 and Table S4a-b).

**Table 1:** Main findings of studies approaching the role of the NPS system in learning and memory

Ref.	Study	Model	Memory type	Memory-related key findings
[27]	Bengoetxea et al. 2021	Mouse	Conditioning (aversive)	Expression of the human-specific hypofunctional NPSR1 N107 genotype improves extinction of conditioned fear in both male and female mice.
[19]	Bicakci et al. 2021	Mouse	Episodic memory (spatial), Reversal learning	Ablating *Npsr1* improves acquisition of T-maze discrimination. Nasally administering NPS improves reversal learning.
[28]	Cannella et al. 2009	Rat	Conditioning (appetitive)	Injection of NPS in the cerebral ventricle (i.c.v) or the lateral hypothalamus (i-LH) facilitates reinstatement of alcohol-seeking behavior.
[34]	Cannella et al. 2016	Rat	Conditioning (appetitive)	In alcohol-preferring but not normal rats, NPS reduces alcohol self-administration. In normal but not alcohol-preferring animals, alcohol-seeking behavior reinstatement is facilitated by NPS.
[35]	Cao et al. 2011	Rat	Conditioning (appetitive)	NPS (i.c.v) facilitates retrieval of cue-assisted conditioning and dose-dependently modulates conditioned place preference (CPP).
[36]	Chauveau et al. 2012	Mouse	Conditioning (aversive)	Inhibition of the NPSR1 in the lateral amygdala leads to an increase of conditioned fear responses during extinction under stress pre-exposure, while the receptor’s activation with NPS prevents the stress-preexposure effect and facilitates fear extinction.
[37]	Chou et al. 2021	Mouse	Conditioning (appetitive)	NPS (i.c.v) facilitates reinstatement of extinguished cocaine CPP, while NPSR1 antagonism (i.p.) prevents restrain stress reinstatement of cocaine CPP. This effect seems to be mediated by orexin and endocannabinoid receptors in the ventral tegmental area (VTA).
[38]	Clark et al. 2017	Mouse	Conditioning (aversive)	Either NPS or an analog compound (a truncated version of the NPS peptide) facilitates the consolidation of acquired inhibitory avoidance behavior.
[31]	Costa et al. 2024	Mouse	Conditioning (aversive)	NPS (i.c.v) potentially interacts with the estrous cycle of female mice in modulating inhibitory avoidance memory. Effects are more robust in high estrogen stages.
[39]	Duangdao et al. 2009	Mouse	Sensorimotor gating	*Npsr1*-deficient mice show normal levels of startle habituation and pre-pulse inhibition, but an improved motor skill performance.
[40]	Enquist et al. 2012	Mouse	Conditioning (appetitive, aversive)	NPS (i.c.v) does not induce conditioned place preference nor avoidance.
[41]	Fendt et al. 2010	Mouse	Conditioning (aversive)	Injecting NPS into the basolateral amygdala blocks conditioned fear expression in a dose-dependent manner, without affecting locomotion.
[42]	Fendt et al. 2011	Mouse	Conditioning (aversive), Sensorimotor gating	*Npsr1*-deficient mice show a modest increase of fear conditioning but no different extinction of contextual fear or prepulse inhibition of the startle response.
[43]	Garau et al. 2022	Mouse	Conditioning (appetitive, aversive), Episodic memory (spatial, object)	*Npsr1*-deficiency facilitates fear conditioning with lower intensity footshocks, and recall of conditioned fear at higher intensity shocks. NPS precursor-deficiency leads to an improved acquisition and recall of conditioned fear at lower shock intensities. However, both genotypes display an impaired extinction learning at average shock intensities, but no differences are present in novel object recognition or spatial memory capacities. Injecting an NPS antagonist into the paraventricular nucleus of the thalamus (PVT) of wildtype mice leads to an impaired recall of conditioned fear, whereas injecting NPS rescues this recall deficit in the NPS precursor knock-out mice.
[44]	Germer et al. 2019	Mouse	Conditioning (aversive)	*Npsr1*-deficient mice show an increased freezing in the retention test but an impaired generalization of long-term contextual fear memory.
[45]	Glotzbach -Schoon et al. 2013	Human	Conditioning (aversive)	Participants carrying both *Npsr1* and 5HTTLPR anxiety-risk alleles exhibit higher startle responses to a fear conditioned stimulus, whereas anxiety ratings for that stimulus increase only in the carriers of the AA *Npsr1* polymorphism.
[46]	Guhn et al. 2015	Human	Working memory (aversive)	Participants carrying the *Npsr1* high-risk anxiety allele display an increased functional near-infrared spectroscopy signal in the dorsolateral and medial prefrontal cortex in response to negative pictures, and a decrease of signal to positive pictures, in a high working memory load condition. The patterns of the signal are opposite to that of the low-risk anxiety allele carriers.
[47]	Han et al. 2009	Mouse	Episodic memory (spatial)	NPS facilitates spatial learning and mitigates MK801-induced learning impairments.
[48]	Han et al. 2014	Mouse	Episodic memory (object)	I.c.v. or intra-basolateral amygdala NPS injection facilitates consolidation of object memory, with a beta-adrenergic receptor antagonist blocking this enhancing effect.
[49]	Huang et al. 2023	Rat	Conditioning (appetitive)	The RTI-263 biased NPSR1 agonist (i.c.v.) attenuates cue-induced reinstatement of cocaine seeking but does not affect cue-induced reinstatement of sucrose-seeking or palatable food intake.
[10]	Jüngling et al. 2008	Mouse	Conditioning (aversive)	Inhibiting NPSR1 in the lateral amygdala and basolateral amygdaloid complex impairs conditioned fear extinction, whereas their activation by NPS facilitates extinction.
[50]	Jüngling et al. 2015	Mouse	Conditioning (aversive)	Fear memory retrieval relies on dynorphin and somatostatin-expressing neurons in the centrolateral amygdala that send GABAergic projections to the NPS neurons in the locus coeruleus (LC).
[51]	Kallupi et al. 2010	Rat	Conditioning (appetitive)	NPS (i.c.v.), i-LH, or intra-perifornical (i-PeF) area increases conditioned reinstatement of cocaine-seeking, whereas inhibition of NPSR1 (i.p.) or i-LH reduces it. The facilitatory effects of i-LH NPS on cocaine-seeking reinstatement are abolished by pretreatment with an orexinergic receptor antagonist.
[52]	Kallupi et al. 2013	Rat	Conditioning (appetitive)	NPSR1 antagonism - i.p., i-LH or i-PeF, but not in the central amygdala or i.c.v., leads to an impairment of cocaine-seeking reinstatement. A high dosage also impairs food-seeking retrieval.
[53]	Kawade et al. 2022	Rat	Conditioning (aversive)	Intra-VTA NPSR1 antagonism impairs, while NPS action facilitates conditioned fear extinction. NPS treatment and fear extinction training increase dopamine efflux and c-Fos expression in the nucleus accumbens shell.
[29]	Kolodziejczyk and Fendt 2020	Mouse	Conditioning (aversive)	*Npsr1*-deficient mice, treated with a high dose of corticosterone, show an impaired contextual fear memory consolidation, a generalization of this memory, and a decreased startle response.
[54]	Kolodziejczyk et al. 2020	Mouse	Episodic memory (social), Conditioning (aversive)	Social novelty recognition is impaired in heterozygous *Npsr1*-deficient females. The conditioned social fear extinction is also impaired in the heterozygous, but facilitated in the homozygous *Npsr1*-deficient mice.
[55]	Kreutzmann et al. 2020	Mouse	Conditioning (aversive), Safety learning	*Npsr1*-deficiency in non-stressed male mice leads to an enhanced safety learning, but the learning-enhancing effect of pre-exposure to electric stimuli is prevented in these mice.
[56]	Li et al. 2009	Mouse	Conditioning (appetitive)	NPS (i.c.v.) does not induce place preference nor aversion, but in higher doses it blocks the morphine-induced CPP.
[57]	Li et al. 2022	Mouse	Motor memory	NPS (i.c.v.) facilitates consolidation of motor memory and leads to learning-associated dendritic spine changes in the motor cortex.
[58]	Li et al. 2025	Rat	Conditioning (appetitive, aversive)	NPS (i.c.v.) facilitates conditioned fear extinction in female alcohol preferring rats. NPS reduces alcohol self-administration in both sexes but does not exacerbate yohimbine-induced reinstatement of alcohol-seeking behavior.
[21]	Liu et al. 2017	Mouse	Conditioning (aversive)	NPS precursor knock-out mice show impaired long-term inhibitory avoidance memory.
[22]	Lukas & Neumann 2012	Rat	Episodic memory (object, social)	NPS (i.c.v) facilitates consolidation of object memory, but does not affect retrieval of social memory nor social preference. NPS (nasal, but not subcutaneous) facilitates acquisition of object memory, in a dose-dependent manner.
[59]	Meis et al. 2008	Mouse	Conditioning (aversive), Fear generalization	NPS injected *via* intra-endopiriform nucleus impairs contextual conditioned-fear memory, but not auditory cued fear memory.
[23]	Okamura et al. 2011	Mouse	Episodic (object, place, context), Conditioning (aversive)	NPS (i.c.v.) enhances inhibitory avoidance memory consolidation in a dose-dependent manner but not its acquisition or retrieval, and facilitates object memory retrieval. Systemic beta-adrenergic receptor antagonism attenuates the memory enhancing effects of NPS. *Npsr1* knock-out mice show an impaired inhibitory avoidance memory, as well as an altered recognition of a novel object, place or context.
[60]	Pañeda et al. 2009	Mouse	Conditioning (appetitive)	NPS (i.c.v.) reinstates extinguished cocaine-seeking behavior in a dose-dependent manner. This effect is not reproducible in mice with a deficiency of the corticotropin-releasing factor receptor 1 (CRF1) or under CRF1 antagonist treatment.
[61]	Raczka et al. 2010	Human	Conditioning (aversive)	Carriers of the *Npsr1* high-risk anxiety allele show a facilitation of fear conditioning reflected by self-reported fear reactions and functional magnetic resonance imaging signal in the rostral dorsomedial prefrontal cortex, but not in skin conductance responses.
[62]	Rappeneau et al. 2025	Mouse	Episodic memory (spatial, social), Reversal learning	Mice expressing a mutation of the *Npsr1* gene (N107) show an increased performance in rule-reversal learning, indicating enhanced cognitive flexibility.
[63]	Ruzza et al. 2012	Mouse	Episodic memory (object)	No differences are found between the *Npsr1*+/+ and *Npsr1*-/- mice generated in the CD-1 mouse background, in terms of novel object recognition tested at 3 h after the learning trial.
[64]	Sartori et al. 2016	Mouse, Rat	Conditioning (aversive)	NPS (i.c.v., pre-extinction) facilitates conditioned fear extinction in the extinction-deficient 129S1/SvImJ strain of mice. The enhancing effect of NPS on extinction consolidation is potentiated by the co-application with the partial NMDAR agonist D-Cycloserine (pre- or post-extinction learning).
[65]	Schmoutz et al. 2012	Rat	Conditioning (appetitive)	The NPSR1 antagonist SHA68 (i.p.) leads to a decrease of both cocaine- and food-seeking reinstatement, while RTI-118 (i.p.) affects only cocaine-seeking in low dosage, and food-seeking only at high dosage. RTI-118 pretreatment also reduces cue-induced reinstatement of cocaine self-administration (dose-dependent).
[66]	Shao et al. 2016	Mouse	Episodic memory (spatial)	NPS (i.c.v.) rescues both scopolamine- and MK801-impaired retrieval of olfactory spatial memories. NPS treatment increases c-Fos expression in the subiculum complex, an area known to be involved in spatial memory.
[67]	Shirayama et al. 2015	Rat	Conditioning (aversive), Learned helplessness	NPS intra-nucleus accumbens (NAc) shell, but not NAc core, nor bed nucleus of the stria terminalis (BNST) facilitates conditioned avoidance (learned helplessness). The effect is recapitulated by inhibiting NPSR1 (intra-BNST). However, any of these treatments do not affect passive-avoidance memory performance.
[68]	Slattery et al. 2015	Mouse, Rat	Conditioning (aversive)	NPS (i.c.v.) facilitates cued-fear extinction learning in high-anxiety (HAB) rats, and attenuates fear expression in mice and rats carrying this trait. The HAB phenotype is accompanied by lower levels of basal *Npsr1* expression.
[69]	Smith et al. 2014	Mouse	Conditioning (aversive, social)	The mRNA expression of *Nps* is increased following social fear conditioning in submissive mice.
[70]	Thomasson et al. 2017	Mouse	Short-term memory (spatial)	NPS (i.c.v.) alleviates the sleep deprivation effects on the acquisition of short-term spatial memories. This correlates with an increase of the c-Fos expression in the infralimbic cortical neurons.
[71]	Thorsell et al. 2013	Rat	Conditioning (appetitive, aversive)	A novel NPSR1 antagonist (NCGC00185684) induces a decrease in operant alcohol self-administration, without affecting reinstatement following alcohol-seeking extinction.
[72]	Ubaldi et al. 2016	Rat	Conditioning (appetitive)	NPS (i-LH) facilitates discriminative cue-induced reinstatement of alcohol seeking. This effect can be blocked by inhibiting the orexinergic receptors in the PVN or into the BNST, but not into the VTA or the LC.
[73]	Wang et al. 2020	Mouse	Episodic memory (spatial)	NPSR1 antagonists SHA68 (i.p) and (D-Val^5^)NPS (i.c.v.) impair retrieval of spatial olfactory memory.
[17]	Xing et al. 2019	Mouse	Conditioning (aversive)	Mice carrying an *NPSR1* missense mutation (*Npsr1*-Y206H) sleep less but show resilience to the detrimental effects of sleep deprivation on contextual memory consolidation.
[74]	Zhao et al. 2010	Rat	Episodic memory (spatial)	NPS (i.c.v.) mitigates spatial learning and memory impairments caused by 72 h REM sleep deprivation.
[75]	Zhu et al. 2010	Mouse	Episodic memory (spatial), Sensorimotor gating	*Npsr1*-deficient male mice show decreased acoustic startle responses at prepulse display, but no differences are found between genotypes in prepulse inhibition, and spatial memory acquisition or retrieval.
[76]	Zoicas et al. 2016	Mouse	Conditioning (appetitive, aversive)	NPS (i.c.v.) facilitates conditioned social fear extinction and reinstate social preference, in a dose-dependent manner, while an NPSR1 antagonist (D-Cys[^t^Bu]^5^-NPS) does not alter social fear conditioning.

### Qualitative synthesis of the included studies

**Fig. 3:**
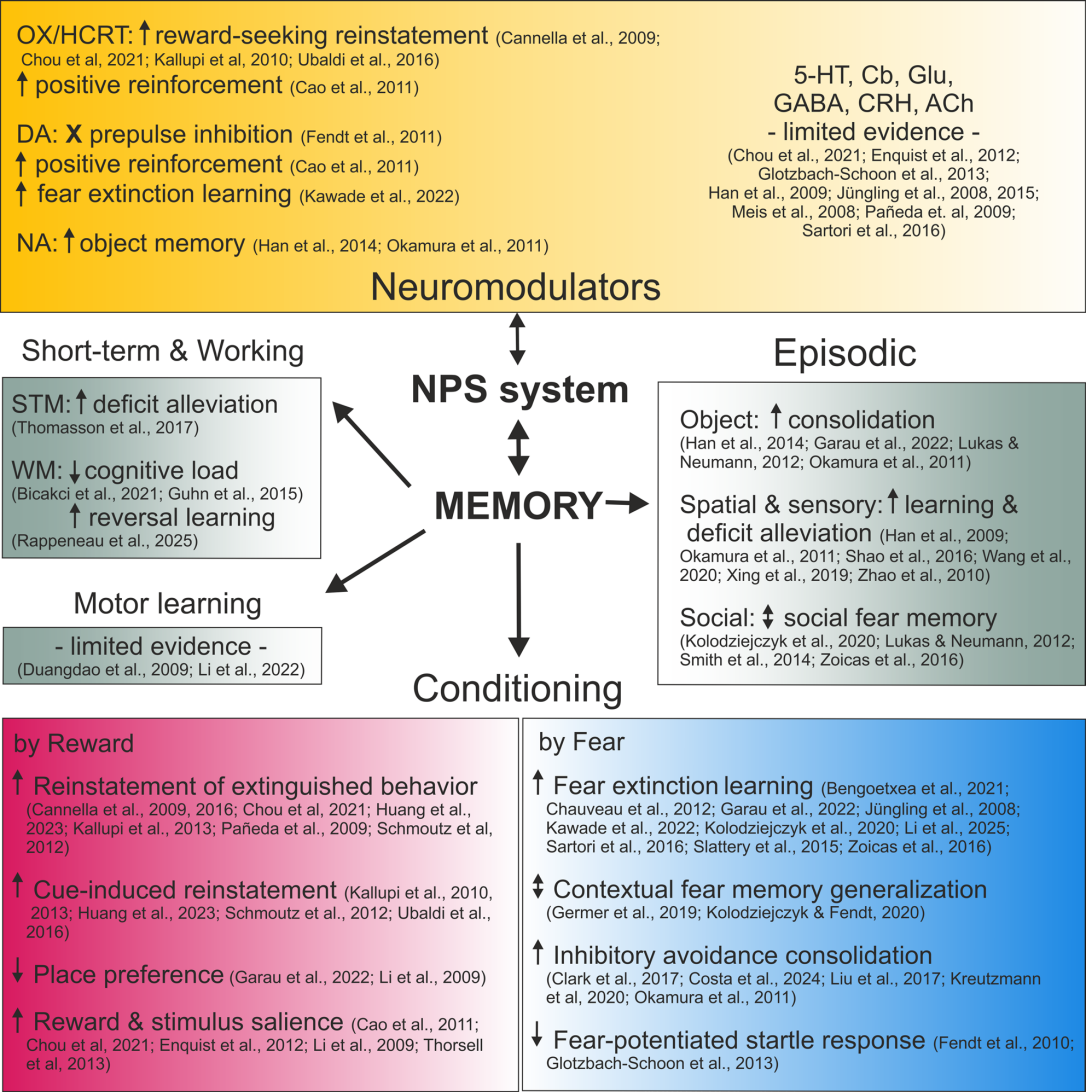
Synthesis of main findings on the influence of the NPS system on memory. OX/HCRT: orexin/hypocretin, DA: dopamine, NA: noradrenaline, 5-HT: serotonin, Cb: endocannabinoid, Glu: glutamate, GABA: gamma-aminobutyric acid, CRH: corticotropin releasing hormone, ACh: acetylcholine, STM: short-term memory, WM: working memory, arrows: up – facilitation, down – attenuation or inhibition, both direction – mixed effects, X sign – no effect

The presence of NPSR1 in key areas for memory, as the paleo- and neocortex, or amygdala [8], suggests the potential modulatory role of the NPS system for mnemonic functions. Since its first characterization [4], the NPS system has been shown to facilitate different forms of memory. In the following, we will present the reported findings on NPS system modulation of memory, grouped by the main type of memory tested. This includes relevant methodological aspects of the studies, a global overview of NPS system action on memory function, and interactions with other modulatory systems (see summarized findings in Fig. 3).

Across memory domains, a unifying feature of the NPS system modulation is its preferential influence on situations requiring the prioritization of learned information, rather than on initial learning itself. NPS effects are most robust during extinction learning, reinstatement, and context-dependent expression of memory, while acquisition and baseline performance are often unaffected.

#### NPS system in short-term and working memory

Although both last for a short period (see Table S2), one important difference between short-term and working memory is that the latter involves not only storing the information but also manipulating it to perform complex cognitive tasks such as reversing the order of the memorized items [77, 78]. How the NPS system modulates working or short-term memory has, thus far, been less investigated.

##### NPS ameliorates short-term memory deficits

Thomasson et al. [70] used a T-maze spontaneous spatial alternation task to test the effect of NPS administration on short-term memory under sleep restriction. Here, it was found that intracerebroventricular (i.c.v.) NPS, administered before alternation testing, overcame the memory deficits induced in mice after 20 h of sleep prevention and that these effects were associated with an increased activation of the infralimbic area, independent of the anxiety-like behavior. The infralimbic cortex contributes to working memory and conditioned learning [79–81], thus, its activation under NPS injection supports the role of the NPS system in memory processes. The preserved anxiolytic effects of the NPS in sleep-deficient mice may help reduce short-term memory impairments caused by sleep restriction [70].

##### NPSR1 modulates working memory under emotional stress

In a study conducted on healthy human volunteers, Guhn et al. [46] tested the genotype effects of the NPS receptor 1 (NPSR1) - AA homozygotes vs. the T allele carriers, on emotional working memory. Although no skin conductance or subjective assessment differences were found between genotypes in performing an n-back task with emotion-eliciting pictures, the activation of the dorsolateral and medial prefrontal cortex in functional near-infrared spectroscopy recordings differed [46]. When processing negative emotion pictures in a high working memory load task, the cortical signal increased in the T allele carriers but decreased in the AA homozygotes [46]. The ancestral T allele (with a higher transmitter functionality vs. the hypofunctional A allele) is overrepresented in panic disorder patients [1, 82, 83]. Healthy patients carrying the T allele demonstrate higher rates of fear conditioning [45, 61] and elevated anxiety sensitivity [84]. These support the idea that an increased activation of the prefrontal cortical areas in the *NPSR1* T allele carriers, during a high-load emotional working memory task, may reflect a compensatory mechanism in the top-down control of emotion-processing in sub-cortical areas facilitated by a hyperactive NPS system [46].

In rodents, two studies looked at cognitive flexibility assessed as reversal learning - either in a spatial working memory task [19], or in the attentional set shifting task requiring association of reward with various sets of odors and digging media [62]. *Npsr1*-/- mice displayed a superior discrimination of the baited arm, but no reversal learning differences in the T-maze, when compared to the *Npsr1*-intact mice [19]. Interestingly, however, nasal administration of NPS in the wildtype (WT) mice led to a facilitation of reversal learning and the use of an allocentric learning strategy, without affecting the discrimination capacity of the animals or their locomotion [19]. Knock-in mice, carrying the human *NPSR1*-N107 genetic variant, were shown to display an increased anxiety behavior without a higher hormonal stress response, and had an enhanced rule-reversal learning in comparison to mice carrying the ancestral murine *NPSR1*-I107 genetic variant [62]. Taken together, these two studies suggest that there is a fine balance between the NPSR1 function and NPS availability. While the complete deficiency of the receptor facilitates acquisition of a simple learning task but no changes in performance in a more complex task requiring cognitive flexibility, a higher activity of the receptor, or a higher availability of NPS, leads to an enhanced performance in solving a complex learning task. This supports the role of NPS as an emotion regulator (i.e., decreased anxiety) for better cognitive control.

#### NPS system in episodic memory

Lasting for more than just a few minutes and up to a lifetime, long-term memory can be classified into* explicit* forms of memory, such as the memory of specific events (episodic) or generalized knowledge (semantic), and* implicit* memory - including forms of learning leading to skills and habits (procedural memory), priming, habituation, and conditioning [85].

The role of NPS in episodic memory was investigated in animal models using paradigms testing the learning and memory of spatial [23, 43, 66, 73–75], social [22], and object information [22, 23, 43, 48, 63].

##### NPS facilitates spatial memory and mitigates spatial memory deficits

The Morris water maze paradigm was mostly used to assess spatial memory under NPS modulation. Here, it was shown that NPS applied i.c.v. facilitates spatial memory in male Kunming mice [47], and mitigates memory impairments induced either by the MK801 N-methyl-D-aspartate (NMDA) receptor pore blocker in mice [47], used as a model of the first episode of psychosis [86], or after 72-h rapid eye-movement(REM) sleep deprivation in rats [74]. Although both studies mentioned the amelioration of spatial memory deficits by NPS in the water maze, only the study using mice reported memory-enhancing results [47]. This could be, however, explained by the lower dosage used in rats [74], 0.5 nmol, compared to 1 nmol used in mice [47]. Furthermore, using an associative spatial memory paradigm, where step-through inhibitory avoidance was tested in mice, Okamura and colleagues showed that post-training central administration of NPS enhanced memory retention dose-dependently [23].

Two other studies employed a spatial memory paradigm where olfaction was used to guide spatial learning in C57BL/6J WT mice [66, 73]. Based on the previous findings on the NPSR1 activity in the olfactory cortex and the hippocampal formation [87], olfactory spatial memory was tested in mice experiencing scopolamine- or MK801-induced memory deficits, and being injected i.c.v. with NPS [66]. The task employed a hole board where four distinct odorants were displayed during the training. At test, the animals were required to discriminate a novel spatial configuration of known odorants [88]. Similar to the experiments where the Morris water maze was used to test spatial memory [47, 74], NPS mitigated the memory deficits in the olfactory spatial memory test [66]. Moreover, the NPS i.c.v. injection led to a selective activation of the NPSR1-expressing neurons, marked by an enhanced c-Fos expression in the subiculum complex of the hippocampal formation, thus supporting the role of the NPS system in facilitating memory function [66].

##### Mild spatial memory impairments are induced by NPS receptor deficiency or inhibition

To test for the effects of NPS system disruption on spatial memory, *Npsr1*-deficient mice were used in two studies employing the Morris water maze [43, 75]. Here, no genotype differences were found in either the visible or hidden platform versions of the task [43, 75]. In the reverse version, however, an increased heading error to the platform was found in the male mice only [75]. Moreover, it was shown that the inhibition of the NPSR1 by either of the two selective antagonists - [D-Val^5^]NPS, i.c.v., or SHA-68, intraperitoneally (i.p.), significantly impaired the olfactory spatial memory test performance [73]. In the same study, *ex vivo* immunolabeling following the olfactory spatial memory test showed a reduced activation of the NPSR1-expressing neurons in the olfactory nucleus, piriform cortex, and hippocampal formation areas of the memory-impaired animals. These support the hypothesis of a modulatory role of the endogenous NPS in olfactory spatial memory [73]. The apparent inconsistencies between the results found in the *Npsr1*-deficient mice tested for spatial memory [43, 75], and those where olfactory spatial memory was tested in pharmacologically blocked NPSR1 animals [66, 73], could be explained by the differences between the transient inhibition of the NPSR1 by pharmacological agents, and the constitutive knock-out of the gene. However, the differences in paradigms used, anatomical regions targeted, and modulatory systems involved, could also account for this.

##### NPS enhances the consolidation of object recognition memory

Novel object recognition task is a common paradigm to test object recognition memory following a brief encoding episode [89]. The task was used to test the role of the NPS system in memory under pharmacological treatment of mice and rats [22, 23, 43, 48], or in genetically modified mice [23, 43, 63].

In one study [23] C57Bl/6 mice explored two identical objects for five minutes, afterwards, 1 nmol NPS or vehicle was injected i.c.v. NPS administration consistently extended object recognition memory beyond time points at which control animals no longer discriminated novel objects [23]. This suggests a memory consolidation enhancement produced by the NPS i.c.v. injection. Similar results were obtained by Lukas and Neumann [22] in adult male Wistar rats.The effects were recapitulated when NPS was administered nasally instead, in a dose-dependent manner (at 4 nmol, but not at 0.4 nmol). However, this was not the case for subcutaneous administration [22]. Using a similar strategy, Han and colleagues [48] recapitulated the results in a Kunming strain of Swiss mice, showing that NPS injected i.c.v. 5 min after training improves 24-h recognition memory, and that these effects involve the noradrenergic system.

NPS memory facilitation effects were also tested in specific brain regions [43, 48]. Bilateral injection in the BLA 5 min after briefly exploring two identical objects led to the recognition of the novel object displayed in the test session 24 h later, indicating that this effect is dependent on the noradrenergic system, as the co-administration of NPS and propranolol abolished the memory facilitation effect [48]. Direct administration of the NPS in the paraventricular nucleus of the thalamus (PVT, 1 pmol) also enhanced recognition memory in WT mice, and rescued recognition in NPS precursor knock-out (KO) mice [43].

Object memory performance of *Npsr1*-deficient mice varies by different strain. Okamura and collaborators [23] reported deficits in *Npsr1* KO mice bred in the 129S6/SvEvTac background at 24 h, while backcrossed mice to CD-1 showed no differences between the *Npsr1+/+* and *Npsr1-/-* genotypes at 3, 24, or 48 h [63]. These variations might stem from differences in locomotor activity and anxiety between strains [63].

##### *Npsr1*-deficiency affects novel place and novel context recognition

Okamura and colleagues [23] tested novel place and novel context recognition in the 129S6/SvEvTac *Npsr1* KO mice, previously reported to display novel object recognition deficits. Here, similar memory deficits were reported: *Npsr1* KO mice failed to recognize the displaced object and to discriminate between two objects, of which one had been previously displayed in a different context [23]. These suggest that NPS is required for forming complex memories, beyond spatial information encoding.

##### NPS system weakly modulates non-aversive social memory

In the social novelty test, during training, an animal encounters an unfamiliar conspecific, while at test, it encounters the same animal together with a novel one. Exploring the new animal indicates recognition capacity together with a propensity towards social novelty. In a study where the social novelty test was used in *Npsr1 +/+*, *+/-*, and *-/-* mice of both sexes, it was shown that, while male mice of all genotypes recognized the novel animal, females failed to do so, with the *Npsr1* heterozygotes female mice also showing a lack of sociability during the training phase [54]. The lack of sociability in *Npsr1* heterozygotes female mice, but not homozygotes, might suggest sex-specific compensatory mechanisms in homozygotes that do not occur in the *Npsr1+/-* female mice [54].

Lukas and Neumann [22] investigated social memory in rats, testing for social discrimination capacity at either 60 or 120 min after the first animal was presented. They found that after 120 min the discrimination capacity faded, so 1 nmol NPS was administered i.c.v. after the acquisition, and rats were tested at that time. No differences were found between NPS- and vehicle-injected rats. This indicates that social memory consolidation is rather not facilitated by NPS.

While there is no obvious NPS effect on social memory in non-aversive conditions [54, 22], animals with prior social defeat experience treated with i.c.v. NPS reversed their aversion for the dominant conspecific, leading to a higher exploration time for the social vs. non-social stimulus [76]. This suggests that NPS system modulation of social memory would be mediated by emotional components and regulated by corresponding brain regions. In agreement, mRNA *Nps* expression was higher in the basolateral and central amygdala in submissive animals that underwent Stress-Alternatives Model testing [69].

#### NPS contribution to fear-elicited learning

##### The study of NPS system in aversive conditioning

Fear is a fundamental emotion essential for our survival, making fear memory one of the most extensively studied memory types. It encompasses the encoding, consolidation, and retrieval phases, as well as the extinction learning of behaviors that help organisms defeat or attenuate fear [47, 90]. We refer here to extinction as a distinct learning process in which a new memory is formed, indicating that the conditioned stimulus no longer predicts the unconditioned stimulus. Experimental paradigms based on associative learning are widely used to investigate fear memory. These include classical (Pavlovian) conditioning, such as *contextual fear conditioning*, where an aversive unconditioned stimulus (US), like a shock or an aversion inducing drug, is paired with a distinct environment (conditioned stimulus, CS), and *cue-induced fear conditioning*, where the US is associated with an auditory tone or a visual cue (CS). *Instrumental (operant) conditioning* paradigms, notably inhibitory avoidance, are also commonly used [90]. Additionally, fear-potentiated startle and prepulse inhibition tasks assess fear-related memory with a focus on sensorimotor gating [32].

A persistent difficulty in extinguishing learned fear is a hallmark of anxiety disorders, including panic disorder, phobias, and trauma-related conditions. Abnormal fear-learning mechanisms likely contribute to their development and persistence [91]. Most anxiety disorders are more frequent in women [92], and recent findings have shown that the estrous cycle influences NPS-mediated behaviors [31]. Therefore, understanding the mechanisms underlying fear extinction learning and the role of the neuropeptide S (NPS) system in this process may offer insights into new therapeutic strategies.

The use of fear-based tasks under pharmacological manipulation provides a valuable tool for developing treatments targeting anxiety and stress-related symptoms [90]. It was shown that the NPS system is also relevant in this context: its activation promotes arousal [93], wakefulness [94], and anxiolytic-like behaviors [4]. NPS receptor and NPS precursor KO mice exhibited mildly increased anxiety-like behaviors in tests measuring responses to stress and novelty [21]. Interestingly, heterozygous littermates presented behavioral deficits similar to NPS−/− mice or displayed intermediate phenotypes [21], suggesting limited ligand availability in critical neural circuits.

##### NPS facilitates fear-extinction learning in Pavlovian conditioning

In studying the influence of the NPS system on Pavlovian fear conditioning, the main differences were observed during extinction learning. The importance of NPSR1 genetic variants for fear-memory extinction was highlighted in both animal and human experiments. In healthy volunteers, it was shown that the carriers of a high anxiety risk allele of the *NPSR1* gene (T+) extinguished conditioned fear earlier than the non-risk allele (AA) group [45]. Moreover, the T+ carriers tend to have smaller skin reaction conductance scores in the extinction learning than the no-risk allele carriers [61]. In this line, experiments conducted in “humanized” transgenic mice revealed the influence of the *Npsr1* polymorphisms in fear extinction circuits in the amygdala, in close interaction with other factors, such as the degree of stimulus salience and sex [27]. Here, the mice expressing a human *NPSR1* genetic variant, associated with increased anxiety sensitivity and panic disorder risk, have shown an improved fear extinction learning after intra-amygdala injection of an NPSR1 antagonist, in a similar fashion to the mice where a distinct *Npsr1* variant, known to be protective against excessive anxiety, was expressed [27]. This suggests that the NPS system mediates the interaction between stress levels and fear extinction learning capabilities.

In a social fear conditioning paradigm, *Npsr1*-deficient mice display normal acquisition and expression of conditioned fear, but a genotype-dependent difference in the extinction process, with the heterozygous mice being impaired and the homozygous mice being facilitated in extinguishing social fear [54].

The impact of the NPS system on fear memory was examined in animals with high anxiety predisposition [64, 68]. Selectively bred laboratory rodents (mice and rats) showed lower *Npsr1* mRNA expression and multiple polymorphisms compared to normal-anxiety rodents. In high-anxiety animals, NPS elicited a stronger dose-dependent anxiolytic effect. Notably, administering 1 nmol of NPS before extinction learning training completely blocked cue-conditioned fear at 24 h in high-anxiety mice and significantly facilitated it in high-anxiety rats [68].

Continuing on this line of research, it was found that the pretreatment with D-Cycloserine (DCS, i.p) - a partial agonist of the NMDA receptor- improved the cued-conditioned fear extinction retrieval scores in high-anxiety rats with impaired extinction memory [64]. Moreover, when applied at the end of the extinction learning session, in extinction-impaired mice (129S1/SvImJ or S1), DCS facilitated the retrieval of extinction learning at 24-h but not after 13 days, and in weak but not standard conditioning. In the same study, NPS pretreatment before the extinction memory process led to improved extinction scores in the 24-h but not the 13-day retrieval test, whereas pretreatment with NPS combined with DCS administered post-extinction improved the fear extinction scores at both time points. These suggest an interplay between NPSR1 activation early in extinction learning, with an NMDA activation at the beginning of extinction consolidation, resulting in longer-lasting fear extinction memories that would prevent fear reinstatement [64].

When a high dose of NPS (10–50 nmol, i.c.v.) was applied in CD1 WT mice, it facilitated the extinction learning of fear induced by an unfamiliar conspecific under social fear conditioning [76]. Using an NPSR1 antagonist led to no effects on social fear extinction in these animals. In the same study, a cued fear-conditioning paradigm was also employed. Here, a low dose of NPS led to a significant within-session reduction of the conditioned response during extinction training, whereas a high dose reduced its expression for the whole training session [76].

Following this line, Chauveau et al. [36] demonstrated that NPS injected into the lateral amygdala prevented stress-induced hyperexcitability and enhanced fear extinction learning in an auditory fear conditioning paradigm, suggesting its potential to regulate fear responses [36]. Additionally, it was found that NPS facilitates fear extinction when injected directly into the amygdala [10]. At the cellular level, the same authors reported that NPS increases the glutamatergic transmission to intercalated GABAergic neurons in the amygdala, offering a potential pharmacological avenue for anxiety disorder treatments [10]. When NPS was i.c.v. administered after the (retrieval) test, it facilitates extinction learning only in female but not in male alcohol-seeking rats [58].

In a contextual fear-conditioning paradigm, intra-ventral tegmental area (VTA) NPS injection in rats facilitated extinction, whereas the selective NPS antagonist SHA-68 had the opposite effect, suggesting that NPS facilitates extinction learning by modulating the mesolimbic dopaminergic circuit [53]. This is supported by the increased dopamine efflux and cFos expression in the shell of the nucleus accumbens (NAc) of the NPS-treated rats that received fear extinction training [53]. Using a similar methodological approach, Garau and collaborators [43] demonstrated the involvement of the NPS system in encoding stimulus salience by signaling in the PVT, which influences fear extinction and memory formation. Similar salience signaling effects in fear conditioning were also reported when NPS was injected in the endopiriform nucleus (EPN) [59]. Together, these findings indicate that NPS does not uniformly suppresses or enhances fear memories but instead modulates how strongly fear-related cues influence behavior depending on contextual and internal state variables. By biasing extinction learning, contextual discrimination, and generalization, without consistently altering acquisition, the NPS system appears to regulate the significance of aversive memories attenuating their impact on behaviour when the environment requires adaptation.

Concluding on this set of findings, it is interesting to understand why both *Npsr1*-deficiency and NPSR1 activation via NPS administration - either centrally, in amygdala or VTA, lead to the facilitation of conditioned fear extinction learning and memory. The explanation probably resides in the role the NPS system plays in regulating anxiety and its interaction with other anxiety-related neuromodulators [15], but more investigation is needed to better understand the mechanisms behind that.

##### NPS induces mild or no alterations in the acquisition of conditioned fear

Genetic polymorphisms of the *NPSR1* gene predispose to enhanced fear conditioning expression. For example, the carriers of the *NPSR1* T+ allele evaluated their fear reactions as more pronounced at the display of conditioned stimuli, compared to the non-risk allele carriers in a cued-conditioned fear conditioning task [61]. Moreover, in a contextual conditioning paradigm conducted in a virtual environment, the carriers of two polymorphisms with increased anxiety risk genes coding for the 5-HT transporter (S+ allele) and the NPS receptor (T+), respectively, displayed a heightened conditioned fear expression as recorded in the amplitude of their startle response [45]. In line with this, in transgenic mice expressing either anxiety-protective or anxiety-facilitating humanized polymorphisms of the *NPSR1* gene, Bengoetxea et al. [27] did not observe genotype differences in fear conditioning expression but in the extinction learning performance only [27].

For the cued fear conditioning no genetic or pharmacological manipulation differences were found [10, 29, 42, 43, 59, 76]. In the case of contextual fear conditioning, in *Npsr1* KO mice, no change was reported after one-trial fear conditioning [44], or only a weak increase in the retrieval [42], or when corticosterone was pretreated immediately after training [29]. However, others reported no effects on conditioning when either NPS alone [53] or its combination with pre-administered stress was applied before contextual conditioning [36]. No effects on contextual conditioning were found either in transgenic mice with *Npsr1* mutations linked to reduced sleep duration [17]. However, a low dose of NPS, injected in the mouse EPN, reduced the response at retrieval without affecting anxiety levels [59]. This effect is considered to be mediated by an EPN-BLA circuit involved in the salience encoding of contextual factors [59].

The conditioned place avoidance under NPSR1 influence was also tested in mice and rats. In a conditioned place preference task, neither alcohol-naïve nor alcohol-drinking groups showed differences after receiving either vehicle or NPS [40]. Cao et al. [35] reported that NPS was rather appetitive in a similar task. In a drug-induced contextual conditioning procedure in WT rats, pre-treatment with either an NPSR1 antagonist or vehicle was paired with a treatment of the aversion-inducing drug lithium chloride. No contextual aversion was observed to have been induced by the NPSR1 ligand, but rather a mild place preference [71].

##### NPS influence on conditioned fear memory generalization

The generalization of fear memory is a mechanism ensuring fear response flexibility to novel situations, whereas its overgeneralization is a common trait in anxiety disorders [95]. When investigating the role of NPS in modulating these phenomena, it was found that one-trial contextual fear conditioning, which becomes unspecific after one month, was impaired in both *Npsr1-/-* and *Npsr1+/-* mice [44]. The overgeneralization of fear memory was specific at one-week timepoint but became unspecific one-month post-training [44]. Context discrimination was measured as a readout for fear memory generalization specificity, one day, or one month after contextual fear conditioning [29]. Although there were no genotype differences in the context discrimination, a dose of 5 mg/kg of cortisol decreased fear memory specificity and strength at one-month timepoint in the homozygous *Npsr1*-deficient mice only, suggesting a corticosterone-NPS interplay in the generalization of contextual fear memory [29].

A decrease in freezing and risk assessment behavior was seen in male C57BL/6BomTac mice at two-weeks retrieval, tested for fear expression under pretreatment with a dose of 0.1 nmol in the EPN [59]. However, no NPS-induced effect on the generalization of the cued fear conditioning was recorded [59].

Altogether, the findings on the NPS influence on conditioned fear acquisition, corroborated with those on fear generalization, support the hypothesis that NPS has a stronger effect when interfering with contextual rather than cued fear conditioning. Interestingly, a reversed relationship can be seen for the NPS influence on fear extinction memory.

##### The consolidation of inhibitory avoidance learning is enhanced *via* NPSR1

The inhibitory avoidance (IA) paradigm has been used extensively to study the mechanisms of aversive learning and memory, and the effects of drugs to manipulate these mechanisms [96]. In *Npsr1*-deficient mice, immediate and delayed post-training central NPS administration dose-dependently enhanced memory retention in mice, indicating that NPS may act during the consolidation phase to enhance long-term memory [23]. Liu et al. [21] created NPS-precursor KO mice, finding that these mice show significant long-term memory impairments in the IA paradigm compared to WT and heterozygous groups, although they can still form weak aversive memories. These studies support the idea that NPSR1 is essential for aversive memory consolidation. However, the action site of NPS in this learning process does not seem to involve brain regions where NPSR1 has key anxiolytic functions, such as the NAc and the bed nucleus of the stria terminalis (BNST) [67].

A novel NPSR1 agonist demonstrated anxiolytic and memory-facilitating effects similar to NPS but with reduced hyperlocomotion in C57/Bl6 wildtype mice [38]. In the IA paradigm, it produced dose-dependent memory facilitation at the 48-hour test when administered 20 min post-training. The authors propose that arousal related to hyperlocomotion is linked to cAMP production, while anxiolytic and memory effects are associated with calcium mobilization [38].

The anxiolytic effects of NPS facilitating IA performance were also tested in low or high estrogen level female mice [31]. Here, two sets of experiments led to rather contradicting results. In the first round of IA testing, NPS given at 0.1 or 1 nmol 20 min post-training, increased the IA latencies in the low- but not high-estrogen female mice in the 48-h test, with an anxiety reduction demonstrated in the high-estrogen group as reflected in the marble-burying behavior and the amplitude of the startle response [31]. However, the IA memory enhancing effect was not recapitulated in a second set of experiments, with long-lasting effects of previous multiple NPS injections being considered as a confounding factor. Therefore, more investigation needs to be performed on the role of NPS in memory under estrous cycle variations to elucidate this potential interaction.

Similar to extinction learning and IA, safety learning is a mnemonic ability that consists of recognizing and appropiately responding to protective cues againgst danger [97]. In this task, rodents learn to associate a safety cue with the absence of an aversive stimulus, and thus to inhibit the associated fear responses [55]. While in C57Bl/6 wildtype and *Npsr*1-nondeficient mice, electric stimuli-induced stress enhanced safety learning in males, no effect was shown following stressful immobility in either of the WT sex groups [55]. Interestingly, the male *Npsr1*-deficient homozygous mice did not demonstrate safety learning enhancement following stress, but their inhibition scores were rather elevated in the non-stress conditions, and significantly higher than in the *Npsr1*-nondeficient mice in the same experimental conditions [55]. The *Npsr1*-deficient female mice did not differ in their safety learning scores across stress exposure conditions and genotypes. Altogether, these findings support that NPSR1 is involved, at least in male mice, in regulating safety behavior motivated by prior stressful conditions [55].

##### NPS attenuates the fear-potentiated startle response

Another type of fear conditioning, and quite easy to induce, is fear-potentiated startle. When faced with frightening situations, humans and animals can exhibit a common reaction known as the startle reflex. This reflex can also be triggered by neutral stimuli such as a tone or a light, which can then become associated with fear through conditioning [42, 44].

In humans, the startle reflex test has become standard for anxiety related to fear and it has been proven to increase in patients suffering from fear disorders or other conditions that increase their propensity to suffer fear [98, 99]. A functional variant within the *NPSR1* gene leads to an amino-acid exchange (rs324981, Asn^107^Ile) resulting in a gain-of-function in the Ile^107^ variant, which has been associated with panic disorders [1, 82]. Schizophrenia patients carrying the Ile^107^ variant showed significantly reduced startle amplitudes but unaffected prepulse inhibition and habituation as compared with the WT Asn variant subjects [100]. Moreover, the fear-potentiated startle response was found to correlate with the interaction between two genetic polymorphisms of the serotonergic and NPS receptors, associated with increased risk for anxiety [45].

Reports using *Npsr1*-deficient mice for testing the influence of the NPS system on startle response amplitude led to rather contradictory findings. One study found that *Npsr1* deficiency diminishes the acoustic startle response [42], while another showed that this is only the case for male mice [75], or for the heterozygous mice, irrespective of sex [44]. In contrast, Duangdao and collaborators [39] reported no alterations in the acoustic startle response. Procedural differences could, however, account for the opposing results. For example, in the study where no genotype effects were found, the animals experienced no prior stressful procedure, with the startle stimuli being displayed after an 8-min acclimation period with 65 dB background noise [39]. In the experiments with a decreased startle response in the *Npsr1* KO, an i.p. injection and only a 5-min acclimation time, with no background noise, were provided. This potentially primed the WT animals in reacting to acute stress [42]. This speculation is supported by the similarity of the values recorded in transgenic animals [42] to those obtained from both genotypes [39]. Moreover, in the experiments where either heterozygous [44], or male *Npsr1*-deficient mice [75] displayed a reduced startle response amplitude, the same animals had been recently pre-exposed to tests evaluating anxiety-like behaviors. Nonetheless, all these studies reported a lack of effect of the genotype on prepulse inhibition [39, 42, 75]. Additionally, at least two different knockout lines were used in these experiments, which could have influenced the results. Fendt et al. [42], Germer et al. [44], and Duangdao et al. [39] used the same *Npsr1* knockout line based on the first one described by Allen and collaborators [101], while Zhu et al. [75] created their own line (See Table S4a).

Interestingly, 1 nmol NPS injected intra-amygdala in WT mice reduced the conditioned fear-potentiated startle response, leveling it to the response induced by the startle tone alone [41]. The fear responses were not affected by the NPS effects on locomotion. These findings indicate an inhibition of the fear-conditioned response due to NPSR1 action in the amygdala.

Looking further into the interaction of NPS with other stress-regulating mechanisms, *Npsr1*-deficient mice that underwent fear-conditioning, were injected with corticosterone and tested for the startle response one hour and one day post-treatment. In these experiments, the homozygous animals treated with 5 mg/kg corticosterone displayed an attenuated fear-potentiated startle response, only for the high stimulus intensities [29]. This supports the hypothesis that an interaction between corticosterone and the NPS system regulates the stress response. Moreover, when corroborated with the effects found in the study where NPS was injected in the amygdala [41], it suggests that a certain level of stress needs to be in place so that NPS can have ameliorating effects on the fear-potentiated startle response.

##### The anxiolytic effects of NPS modulate social fear memory

Being cautious of unfamiliar conspecifics is natural, as these can transmit diseases or be aggressive. However, when this caution is prolonged or excessive, causing fear of any social exposure, it may result in social anxiety disorder. Researchers have found that some anxiety disorders in humans are linked to polymorphisms of the *NPSR1* [102]. Moreover, the *NPSR1* gene has been associated with an increase in the cortisol response to social stress [103]. In rodents, Kolodziejczyk and collaborators [54] revealed sex-dependent effects on sociability and social fear extinction learning linked to *Npsr1* deficiency, where only the heterozygous female, but not male *Npsr1*-deficient mice, displayed reduced sociability. While *Npsr1* deficiency did not affect conditioned social fear in the acquisition and retrieval phases, it did impair extinction learning in the heterozygous mice and facilitated it in the homozygous animals. No gender differences within the genotypes were investigated for this experiment [54].

Making use of a stressful social decision-making model for mice, Smith et al. [69] evaluated two types of behaviors - escape and submission-, under the encounter of a larger and aggressive conspecific associated with an auditory conditioned stimulus. Classic fear conditioning was observed in the submissive animals together with an increased mRNA expression of *Nps*, a decreased expression of brain-derived neurotrophic factor (BDNF), and an increased corticosterone level in the BLA. The greatest *Nps* mRNA expression level was recorded in the central amygdala, and it was also high in animals exposed to a non-threatening animal, but not in the escaping animals and cage controls. Altogether, these results suggest that NPSR1 activity in the amygdala mediates a learned response to social and contextual stress factors [69].

Using pharmacological manipulations in mice, it has been demonstrated that the anxiolytic effects of NPS contribute to reversing social fear and avoidance behaviors induced by social defeat and fear conditioning [76]. However, in rats, Lukas and Neumann [22] showed that NPS does not facilitate social memory consolidation, in contrast with the effects it had on non-social object memory. Moreover, in the same study, the social preference task after treatment with either NPS or the [D-Cys(^t^Bu)^5^]NPS antagonist was unaffected. Nonetheless, NPS did reduce non-social anxiety in the same group of animals, as shown in the elevated plus-maze test [22].

The reported contrasting results on social fear memory could be explained by the use of different species (mice vs. rats), the intensity of the social fear stimulus (a simple exploration of the social stimulus vs. its association with an aversive footshock), and the NPS dosage ( i.e., Zoicas et al. [76] - obtained ameliorating effects at 10 and 50 nmol). Therefore, further experiments using different paradigms must be performed to obtain more clarity on this matter.

#### NPS contribution to reward-induced conditioning

##### The NPS system is not essential for reward reinforcement in operant conditioning

NPS system influence on learning was tested in behavioral paradigms using operant training of drug self-administration in rodents [28, 34, 35, 49, 51, 52, 60, 65, 71, 72]. This is a classical model used to better understand reward-motivated behavior and in finding treatments for addiction [104]. The method comprises three main stages [28, 52, 65, 71]. During *conditioning* , the animals undergo operant training to learn to associate lever pressing with sensory cues indicating the availability of a reward, usually a liquid drug administered i.c.v. or intravenous (i.v.), or an edible food. Once the animals reach a baseline level of successful self-administration of the reward, *extinction learning* begins. Here, no cues or rewards are provided, leading to a drastic reduction in the motivation of the animal to press the lever. Finally, the *reinstatement* testing consists of a single session, similar to the conditioning stage except that the drug or its control is not provided.

NPS or NPSR1 antagonists exerted minimal and inconsistent effects on baseline reward acquisition and maintenance [28, 34, 51, 52, 65, 71], supporting the conclusion that the NPS system is not required for primary reinforcement learning. Doses of up to 2 nmol NPS i.c.v. did not affect baseline self-administration nor inactive lever presses in rats conditioned to self-administer cocaine [51] or alcohol [28]. However, the NPS treatment decreased the reinforced responses in an alcohol-preferring rat strain (Marchigian Sardinian), presumably due to the anxiolytic effects of NPS [34]. NPSR1 antagonists were tested to see how inhibiting NPSR1 affects previously acquired operant conditioning, with reports showing contrasting results.

On the one hand, no effects were recorded on cocaine-conditioned self-administration, nor inactive lever presses, after administering NPSR1 antagonists [51, 52]. However, the NPSR1-QA1 antagonist did reduce food self-administration at its highest dose [52], SHA-68 induced a dose-dependent decrease of both cocaine and food intake, and the RTI-118 only affected cocaine, but not food intake, at lower doses [65].

On the other hand, NCGC00185684, a biased NPSR1 antagonist, decreased operant alcohol self-administration and lowered the motivation for alcohol reward [71]. This could be first explained by the use of a high dose, relative to the potency of the drug, considering that, for example, SHA-68 at 30 mg/kg did not affect cocaine self-administration [51] but did so at 50 kg/mg [65]. Secondly, this can also suggest an effect of the dosage on motivation as suggested by the inhibition of progressive ratio response [71]. Altogether, these findings rather support that the NPS system is not required for reward-seeking operant conditioning but can alter the motivation factor in this process.

##### NPS facilitates reinstatement of extinguished reward-seeking behavior

Three studies examined the role of NPS in reinstating conditioned responses without a reinstatement stimulus. Cannella and collaborators [34] found that NPS doses of up to 2 nmol increase lever presses during extinction learning in Wistar rats but, interestingly, not in alcohol-preferring rats, potentially due to a priming-like effect of NPS that is active in alcohol preference-neutral rats. The alcohol-preferring animals show higher anxiety levels and putatively use the NPS effect to reduce that stress but not to enhance motivation to seek reward. In another study, Pañeda and collaborators [60] showed that NPS i.c.v. injection (0.45 nmol) was able to potently reinstate the drug-seeking behavior, without increasing the inactive lever presses. In line with these findings, the treatment of Wistar rats with an NPSR1 antagonist reduced lever pressing at extinction learning, in a dose-dependent manner [52]. However, confounding effects from locomotor function disruption were not ruled out in the higher dose effects on extinction responding.

##### NPS facilitates cue-induced reinstatement of reward-seeking behavior

The reinstatement of conditioned drug-seeking in rodents is a classical paradigm used to model relapse experiences in humans [105]. Reinstatement can be induced by using the same sensorial cue paired with the drug availability during the acquisition, or other stimuli such as priming with addictive drugs or stressful experiences (i.e., [71]). As mentioned above, when administered without using cues to elicit the drug-seeking behavior, NPS leads to a reinstatement [52, 60]. However, NPS has also been shown to interact with other factors that induce drug-seeking reinstatement [28, 34, 49, 51, 52, 65, 72].

During the reinstatement test, the increase in the number of only the active lever presses, when the drug-associated cue is active, indicates the facilitation of drug-seeking behavior relapse. NPS was pretreated before testing for reinstatement of reward-seeking behavior for various drugs or foods. For example, three days after a control session of alcohol-seeking reinstatement, NPS was injected i.c.v., and lever presses were counted when either alcohol or water-associated sensory cues were present [28]. In this study, just 2 and 4 nmol NPS pretreatment, and only in sessions where the alcohol-associated cue was present, led to a significant increase in the drug-seeking responses [28]. A similar pro-reinstatement effect was noticed when a repeated relapse model was used instead, with one group being treated with saline and another with NPS in every two of the six reinstatement testing sessions [28].

Intraventricular injections of NPS facilitated as well the reinstatement of conditioned cocaine-seeking induced by the cocaine-, but not water-associated cue, whereas the NPSR1 antagonist SHA-68 injected i.p. significantly inhibited the cocaine-associated cue-induced reinstatement [51]. Confirming the selective action of NPS, the NPSR1 antagonist NPSR1-QA1 (i.p) prior to the reinstatement testing decreased cue-induced cocaine-seeking reinstatement [52].

Similar effects were seen when 0.5 nmol NPS was injected into the lateral hypothalamus (LH), an effect that was further blocked by an i.p. injection with 10 mg/kg of the selective Hcrt-1/Ox-A receptor antagonist SB334867 that did not affect cue-induced reinstatement by itself [28]. The NPS facilitating effects on cue-induced reinstatement were recapitulated in Wistar, but not in the alcohol-preferring rats (the Marchigian Sardinian strain), where NPS failed to affect reinstatement responses and inhibit alcohol self-administration during the conditioning stage [34].

Another study using bilateral injection of NPS intra-LH reported significantly enhanced cue-induced alcohol-seeking in rats [72]. Investigating this further, the authors found that the intra-LH NPS pro-reinstatement effect is being blocked by infusing the Hcrt-1/Ox-A receptor antagonist SB-334867 in the PVN and BNST, but not in the LC and VTA [72]. This indicates that PVN and BNST are sites of action for facilitating the influence of the NPS-orexinergic interaction in cue-induced reinstatement of conditioned drug-seeking [72]. This was also supported by the increasing reinstatement responses in rats where Orexin A was administered either in the PVN or BNST, although with a weaker effect compared to the reinstatement produced by NPS infusion into the LH [72].

Based on the c-Fos immunoreactivity following NPS injection, Kallupi and collaborators [51] decided to inject NPS also in the LH, PeF, DMH, and CeA. Injections of 0.5 nmol NPS in the LH and PeF, but not DMH and CeA, increased the cue-induced cocaine-seeking reinstatement responses. However, only a higher dose (2 nmol) in the DMH induced a similar effect [51]. As the three areas where NPS produced a reinstatement facilitatory effect contain an important part of the orexinergic projection neurons, the hypothesis of an NPS-orexinergic system interaction in drug-seeking reinstatement was further tested. In this regard, it was shown that the infusion with 30 nmol of [D-Cys(^t^Bu)^5^]NPS NPSR1 antagonist in the LH decreased cue-induced reinstatement. The same NPSR1 antagonist had no effect on cue-induced reinstatement when it was infused in the ventricle or injected into the CeA bilaterally, but decreased reinstatement when injected into the PeF and the LH [52]. Moreover, the selective Hcrt-1/Ox-A receptor antagonist SB-334867, injected i.p. prior to infusing NPS into the LH, completely prevented the pro-reinstatement effects of NPS, without affecting reinstatement by itself [51].

To further test the specificity of the NPS effects, various NPSR1 antagonists were employed. For example, it was shown that the NPSR1 antagonist RTI-118 (i.c.v) decreases cue-induced reinstatement of cocaine-seeking in male and female rats, without affecting sucrose pellet seeking reinstatement [49]. In another study, pretreatment with the NPSR1 antagonist RTI-118 (i.p.) decreased the response of reinstatement of cocaine-seeking, and inhibited the priming effect of cocaine and yohimbine in inducing reinstatement of extinguished cocaine-seeking behavior [65]. However, the biased agonist RTI-263 (i.c.v.) also induced a decrease in the cue-induced reinstatement response, without replicating the inhibitory effects of NPS on locomotion and food consumption [49]. Without disregarding the potential benefits, this suggests that comparisons between findings obtained with endogenous NPS or synthetic NPSR1 agonists should be approached cautiously.

##### NPS inhibits reward-associated place preference conditioning but facilitates reinstatement

In its classical form, the conditioned place preference (CPP) paradigm includes: a *preconditioning* stage - in which the animals are allowed to move between two chambers with distinct contextual cues (i.e., wall color, floor texture, olfactory stimuli); an *acquisition* stage, consisting of few consecutive sessions during which the animal is placed alternatively in the chambers paired with either drug or vehicle pretreatment; and, finally, a *post-conditioning* test session, in which the animal freely explores between the rooms without receiving the rewarding drug (i.e. [20]), In an extended version, *extinction learning* and *reinstatement* trials are added to the procedure (i.e. [37]), . For extinction learning, the animals are repeatedly treated with the vehicle while being placed in the drug-paired chamber, while in the reinstatement stage, the animals are allowed to explore either of the chambers, without receiving the rewarding drug, but after being pretreated with potentially modulating drugs or other stimulation (i.e., restraint stress) [37].

After ensuring that NPS (i.c.v., up to 10 nmol) in itself does not produce place preference nor aversion in mice, Li et al. have shown that co-injecting (i.c.v.) NPS with morphine before each conditioning session inhibits the acquisition of CPP in a dose-dependent manner [20]. Similarly, another group of mice, that received a high dose of morphine during the acquisition session, and NPS pretreatment before conditioning testing, displayed a significantly reduced place conditioning performance [20]. This finding was supported by experiments comparing between the performance of WT and *Npsr1* KO mice in a morphine-CPP paradigm. Here, it was shown that the *Npsr1* deficiency leads to attenuated conditioning, irrespective of the rewarding drug dosage [43].

Chou et al. induced cocaine-CPP, followed by extinction learning and reinstatement testing [37]. Similarly to the experiments of drug-seeking operant conditioning described above, pretreatment with 1 nmol (i.c.v.) NPS facilitated the reinstatement of CPP at test [37]. This effect was not present in the saline-pretreated mice, indicating that this was a direct consequence of the drug and not of the injection procedure. Moreover, the NPS pro-reinstatement result was prevented by the i.p. injection of the NPSR1 antagonist SHA-68, with the ligand or its vehicle alone not affecting the cocaine-CPP scores during the reinstatement test [37]. The NPS-induced cocaine reinstatement effects were blocked as well by the pretreatment with either a Hcrt-1/Ox-A or a CB1 receptor antagonist, and both when the ligands were applied systemically and bilaterally in the VTA. This supports the NPS role as an upstream modulator of neurons processing reward and further enabling cocaine-seeking reinstatement [37].

##### NPS acts as a modulator of reward and stimulus salience in appetitive conditioning

Knowing that NPS system is associated with reward-seeking behavior [18], and that its receptors are found in reward-associated areas [5, 8], the direct reward-like effects of the NPS were questioned [35]. Supporting this idea are studies reporting that NPS increases dopamine release in the medial prefrontal cortex [106] and the NAc [107]. However, evidence from behavioral experiments is rather contrasting.

Cao et al. [35] reported that rats with intraventricular implanted cannulas were conditioned to self-administer NPS based on place preference under a cue-assisted procedure and in a dose-dependent manner. Both a dopamine D1 receptor antagonist, applied in the early phases of conditioning, and a hypocretin/orexin receptor antagonist, applied before conditioning, led to a decrease in the preference to self-administer NPS at the cue signal. Nonetheless, the dopaminergic ligand induced a decreased locomotion during drug self-administration, possibly influencing the conditioning results. In the same study, NPS was infused in a place conditioning procedure, where animals were subjected to associating contextual cues with two distinct compartments. Surprisingly, the rats displayed different conditioned behavior depending on the NPS dosage: the high dose (1 nmol) induced place preference, while the medium dose (0.1 nmol) induced aversion, and the low dose (0.01 nmol) had no effects [35]. Notably, the higher doses increased locomotion and rearing, suggesting a sensitizing effect of acutely administered NPS, whereas the high dose only induced a decrease in locomotion when applied chronically for four consecutive days [35]. Taken together, these experiments bring some evidence that NPS might have rewarding-like effects, directly interacting with the dopaminergic and orexinergic systems [35]. Nonetheless, different studies – listed below, led to rather contradictory results, while some doubts can also be raised about potential locomotor confounding factors [35] and mode of calculation of statistical differences between the treatment groups.

When up to 10 nmol NPS was infused prior to a CPP procedure in mice, no effects were reported on either place preference or aversion compared to controls [20, 40]. Moreover, Li and collaborators [20] showed that NPS co-injected i.c.v with morphine during acquisition, reduced the CPP score for morphine in a dose-dependent manner, whereas administered immediately before the conditioning test, reduced the time spent in the conditioned preferred place at 0.1 and 1 nmol, and totally blocked the preference at 10 nmol. The results were recapitulated in an experiment where 10 mg/kg of morphine was applied subcutaneously to induce a potent CPP in alcohol-naïve C57Bl/6J mice. Pretreatment with NPS (3 nmol, i.c.v.) during the conditioning sessions failed to induce place preference or aversion in either alcohol-naïve or alcohol-drinking mice that started the conditioning test 18 h after their last alcohol intake [40]. Adding to other results, the authors concluded that, although NPS proved to have increased anxiolytic and antidepressant effects in alcohol-drinking mice, this is not mediated by any rewarding properties [40]. This is, in fact, also supported by the alcohol consumption reduction effects of NPS [40]. Taken together, these two studies suggest that NPS acts on the reward circuits to rather inhibit both acquisition and expression of conditioned preference induced by reward, without eliciting in itself any reward-seeking behaviors [20, 40].

Recent findings offer a new perspective on the role of NPS in regulating reward. Based on experiments in *Npsr1* KO mice demonstrating the importance of NPS in mitigating fear extinction learning and novel object memory impairments, Garau and collaborators [43] hypothesize that NPS signaling is necessary for encoding stimulus salience, especially by recruiting NPSR1-expressing neurons in the PVT. Adding to that, it was shown that the NPS-induced cocaine reinstatement effects can be blocked by the pretreatment with either a Hcrt-1/Ox-A or a CB1 receptor antagonist, applied bilaterally in the VTA, thus supporting the NPS role as an upstream modulator of neurons processing reward in cocaine-seeking reinstatement [37].

Trying to reconcile the two contrasting findings on the rewarding-like properties of NPS, Cannella et al. suggest that the pro-rewarding results can be mainly explained by the cognitive and arousal-enhancing effects of NPS [108]. Moreover, to better understand the mediating role of NPS system in reward-driven behaviors, a closer look at its interaction with other systems, such as the dopaminergic one, must be further explored.

The absence of consistent NPS effects on reward acquisition or baseline self-administration contrasts with its strong facilitation of cue-induced reinstatement and relapse-like behavior. These findings suggest that NPS effects may be particularly evident when reward-associated cues acquire motivational relevance, rather than reflecting a direct reinforcement of reward learning, particularly in states of deprivation, stress, or extinction learning.

#### Potential contribution of NPS system to motor learning

Taking into account the strong evidence for the effects of NPS system in regulating locomotor activity [4, 42], two studies investigated its contribution to motor skills [39, 57], using the rotarod test in mice. In the first study, three sessions of 5 min were conducted on three consecutive days [39]. Latencies to fall were compared between WT and *Npsr1* KO mice. In the second study, an intensive training of 40 trials of 100 s each, totaling minimum of 40 min, was conducted, followed by NPS injection (1 nmol, i.c.v.). After 24-h, a test of 20 trials over more than 20 min was run [57]. Performance was calculated as a percentual improvement at test compared to the training. The reported findings of these studies were contradictory, as both genetic ablation and pharmacological facilitation of NPSR1 resulted in enhanced motor coordination skills [39, 57]. It is, however, difficult to compare these results because of procedural differences and limitations. First, the study by Li and collaborators [57] lacks transparency in the mode of performance calculation: it is not specified if rotation speed and/or fall latency were computed as the performance value. Second, the NPSR1 ligands injection was applied acutely, after the training, and only 24-h before the test. Therefore, potential stress-induced confounding effects cannot be excluded. Third, the authors also report training-induced physiological correlates (dendritic spines modification) in support of the NPS-facilitated learning [57]. However, due to the early age of the animals (around 5 weeks old), developmental changes could have also affected the recordings. Moreover, neither sex nor different genotype factors were taken into account as potential confounds for the results. Lastly, the rotarod study comparing WT and *Npsr1* KO mice [39] did not test the effects of NPSR1 ligands on skill acquisition to confirm the enhancement effects, therefore a clear conclusion on this is, as yet, difficult to draw.

#### NPS interaction with neuromodulator systems involved in memory regulation

In our pool of selected studies, it was reported that the NPS system influence on memory also recruits other modulatory systems, mainly the hypocretin/orexinergic [28, 35, 37, 51, 72], dopaminergic [35, 42, 53], noradrenergic [23, 48], endocannabinoid [37], and serotonergic [45] systems. The NPS system was also found to interact with neurotransmitters, such as glutamate [47, 64], and gamma-aminobutyric acid (GABA) [10, 40, 50, 59].

##### NPS system - orexin interaction: potentiating the reinstatement of a previously acquired drug-seeking behavior

The orexinergic (or hypocretinergic) system is known to be involved in the control of appetite and reward [109, 110], wakefulness and arousal [111], and fear memory and anxiety [, 112, 113].

Anatomical studies indicate an interaction between the NPS and orexinergic system. First, it was found that intraventricular NPS injections led to an enhanced c-Fos expression in the hypothalamic lateral orexinergic neurons [114]. Second, with tracing studies, Ubaldi et al. [72] found that, in mice, hypothalamic Hcrt-1/Ox-A positive neurons colocalize with NPS-containing axons, with more than a third of the orexinergic neurons expressing NPS receptors. Two distinct neuronal pathways were found to connect the orexinergic neurons from the lateral hypothalamus to either the hypothalamic paraventricular nucleus (PVN) or the BNST [72].

Further functional studies suggest that the NPS system plays an upstream modulating role for the orexinergic system, mainly acting on the Hcrt-1/Ox-A receptors [28, 37, 51, 72, 114]. For example, activation of the NPS receptors in the LH facilitated the cue-induced ethanol-seeking relapse in rats, with this effect being mediated by the LH neurons expressing Hcrt-1/Ox-A receptors [28, 72]. Interestingly, the NPS facilitating effect was recapitulated when orexin-A was infused into the PVN and the BNST and blocked by a selective Hcrt-1/Ox-A antagonist [72]. Similarly, NPS-induced cocaine reinstatement was blocked by inhibiting the Hcrt-1/Ox-A receptors with an antagonist infused either centrally, or in the VTA, in mice [37] or into the lateral hypothalamus or prefornical area, in rats [51]. Furthermore, the cue-assisted intraventricular self-administration of NPS decreased in rats when a Hcrt-1/Ox-A antagonist was applied systemically [35].

Taken together, these findings suggest that, under the influence of environmental cues, NPS activates the orexinergic signal and stimulates the relapse of previously acquired reward-seeking behaviors.

##### NPS - dopaminergic systems interaction: reward-like effects of NPS and enhanced fear extinction learning

As a neurotransmitter, dopamine is mainly involved in regulating locomotion [115], reward signaling [115, 116], and learning and memory [117]. NPS stimulates the dopaminergic signal in the prefrontal cortex [106] and NAc [107], thus facilitating the locomotor function [107] and reward-driven behavior [35]. A circuit mediating the NPS influence on the dopaminergic system is *via* the orexinergic lateral hypothalamic projections arriving in the VTA, in close proximity to the local dopamine neurons [118]. Another described circuit contains the NPS neurons in the peri-coerulear area that project to the VTA, where cells were found to express NPS receptors [53].

The influence of NPS on the dopaminergic system, with relevance for learning and memory, was demonstrated in either reward-driven [35], or fear-induced [53] conditioning behavior. Interestingly, Cao and collaborators [35] found that rats can learn to self-administer NPS by pressing a lever due to the reward-like properties of the peptide, with a dopamine D1 receptor antagonist being able to suppress the NPS-induced behavior. Looking further into the mechanisms of NPS influence on the dopaminergic system, it was found that, when injected into the VTA prior to extinction learning, NPS facilitated, whereas an NPSR1 antagonist impaired extinction of fear memory [53]. Moreover, in the intra-VTA NPS-injected rats, the fear extinction training led to an increased dopamine efflux and higher c-Fos expression in the NAc shell [53]. However, the dopaminergic signal itself seems to not influence the role of the NPSR1 in memory, at least for the prepulse inhibition learning behavior of *Npsr1*-deficient mice [42].

Altogether, these findings suggest that NPS enacts the dopaminergic signal necessary to consolidate learning under less stressful conditions.

##### NPS - noradrenergic systems interaction: facilitating a saliency signal for memory consolidation

The noradrenergic system regulates multiple cognitive, emotional, and autonomic functions [119]. A part of the NPS containing neurons is located in close proximity to the locus coeruleus, the main noradrenergic nucleus in the brain [4]. In object memory, as demonstrated in mice, NPS acts on the noradrenergic system to facilitate learning [23, 48]. Here, it was shown that centrally administered NPS can enhance object recognition memory when object learning time is very short [exploration < 5 s] [48], or when object recognition is measured at more than 24 h after a 5-min training session [23]. Interestingly, in both cases, the systemic application of propranolol, a non-selective beta-adrenergic receptor antagonist, blocked the memory enhancing effects [23, 48]. Moreover, Okamura et al. showed that this was also the case for inhibitory avoidance memory, as, while NPS applied post-training increased the time spent in the safe space 48 h after conditioning, the co-administration of NPS with propranolol led to similar latencies as for the controls, these suggesting that the inhibitory avoidance memory consolidation requires an intact noradrenergic system [23].

The NPS-noradrenergic system interaction was further clarified with experiments where it was shown that the NPS-enhancing effects on object memory, triggered centrally, could be replicated when NPS was injected in the BLA, an area known for its role in the consolidation of emotional arousal-modulated memory [120], and also blocked by co-injecting the propranolol antagonist [48]. This is also supported by histological reports of high expression of *Npsr1* mRNA in the BLA [3].

Taken together, these findings suggest that NPS have a facilitatory role in consolidating longer-lasting memories, possibly through interactions with the noradrenergic system [23].

##### NPS system interaction with other neurotransmitter and modulatory systems

Several *in vitro * studies suggest an interaction between NPS and other modulatory systems. For example, it was reported that, at low concentrations, in the mouse brain, NPS acts as a selective inhibitor of evoked serotonin, noradrenaline, and glycine release at the frontal cortex nerve terminals [121, 122], as well as of the release of serotonin and glycine in the amygdala [121].

The interaction of serotonin and NPS systems is also supported by the finding that an acute administration of the selective serotonin reuptake inhibitor escitalopram increases the expression of the *Npsr1* mRNA in the rat brainstem and the hypothalamus [123], an interaction that might be involved in anxiety regulation. Further *in* *vivo* evidence is coming from contextual fear conditioning studies conducted in healthy normal volunteers. Here, it was found that only the carriers of increased anxiety risk alleles, 5HTTLPR (S+) and *NPSR1* (T+) polymorphisms, display an enhanced fear-potentiated startle response in an anxiety-inducing context [45]. Interestingly, the 5HTTLPR (S+) polymorphism had no effect on explicit anxiety ratings, whereas the carriers of only no-risk allele within the *NPSR1* genotype selected higher anxiety ratings in the contextual fear conditioning task, thus indicating that the interaction of the two risk alleles predominantly affects the implicit expression of anxiety responses [45].

Limited evidence for the interaction of NPS with the endocannabinoid system comes from a study showing that NPS reinstated extinguished cocaine CPP behavior, and that this effect was abolished by i.p. or intra-VTA injections of a CB1 receptor antagonist [37]. This supports the hypothesis that the cannabinoid receptors are an important element in the NPS-facilitating effects on drug-seeking relapse. In this regard, under stress conditions, the NPS-containing neurons release the neuropeptide further activating orexinergic neurons that project to the VTA, and from here, together with the cannabinoid receptors, activate the dopaminergic neurons involved in a reward-driven behavior [37, 124].

The interaction between NPS and the corticotropin-releasing hormone (CRH) was particularly reported to affect locomotion [60, 125], stress [126], and addiction [60, 108]. Using an operant conditioning paradigm where WT mice were trained to self-administer cocaine, it was shown that NPS can reinstate drug-seeking behavior in the absence of cocaine. Interestingly, when corticotropin-releasing factor receptor 1 (CRF1) KO mice were used for the same task, NPS could not reinstate cocaine-seeking, although it still had anxiolytic effects similar to those in the WT. The results were recapitulated when using a CRF1 antagonist [60].

Finally, NPS was reported to also play a role in glutamatergic, GABAergic, and cholinergic neurotransmission. For example, the interaction of NPS with the N-methyl-D-aspartate receptor (NMDAR) was reported in *in vivo* studies where it was shown that NPS mitigates spatial memory impairments induced by the MK801 NMDAR antagonist [47], as well as in strengthening extinction memory when NPS was co-applied with the D-cycloserine NMDAR agonist in 129S1/SvImJ extinction-deficient mice [64]. Evidence for an NPS interaction with the GABAergic system was mainly gathered from *ex vivo* studies. In mice that underwent alcohol-seeking conditioning and developed depression, NPS was found to lower depressive behavior and, to potentiate the GABAergic inhibitory postsynaptic current amplitude in the BLA [40]. Moreover, an increased efficacy of the influence elicited by GABAergic neurons from the centrolateral and centromedial amygdala projecting to brainstem NPS neurons, was observed during fear memory retrieval [50]. Furthermore, it was shown that NPS increases glutamatergic transmission of GABAergic interneurons in the BLA [10, 59], *via* an endopiriform nucleus - amygdala circuit that modulates the expression of contextual, but not cue-induced fear memory in mice [59]. For the NPS-cholinergic system interplay, NPS was shown to be able to reverse object recognition memory induced by the nonselective muscarinic antagonist scopolamine in mice [127].

Overall, these results provide evidence that NPS is an important neuromodulator of emotionally-modulated learning and memory function, interacting with many other modulating and transmission systems required for the enactment of various cognitive, affective, and autonomic functions.

## Perspectives and limitations

The NPS system influences the encoding of both aversive [10, 27, 29, 41, 44, 61], neutral [39, 57], and appetitive stimuli [28, 34, 51]. The NPS system in the paraventricular thalamus (PVT) has been proposed to play a crucial role in encoding the salience of stimuli in a manner that is both dose-dependent and reliant on NPS levels [43]. This function is key to memory formation, making this brain structure a strong candidate for modulating fear-based, appetitive, and neutral-content memory. Since the PVT receives one of the highest densities of NPS projections and contains a large population of NPSR1-expressing neurons [3, 93], the question remains if, and how, the PVT NPS system modulates the stimulus valence across various memory types.

We hypothesize that, through PVT projections, the NPS system influences memory formation in key areas such as the amygdala, medial prefrontal cortex (mPFC), and the NAc. Already, PVT projections to the amygdala and mPFC have been reported [128, 129], as well as activation of immediate early genes in these regions following PVT NPSR1 activation [129]. Therefore, the NPS system may contribute to processes related to stimulus salience, as discussed also previously [43].

The discoveries of the role of the NPS system in fear memory support this hypothesis. For example, the extinction process of conditioned fear under *Npsr1* genetic manipulation yielded different results. Mice carrying the human-specific hypofunctional N107 gene, from humans associated with increased anxiety sensitivity, displayed improved extinction learning of conditioned fear [27]. Similarly, KO mice of either *Npsr1* or *Nps* precursor genes displayed an attenuated extinction learning in a stimulus intensity-dependent manner [43], suggesting that NPS signaling may influence the behavioural relevance assigned to fear-related stimuli. Differences between constitutive KO and temporal pharmacological intervention effects have also been reported. *Npsr1* KO mice exhibit enhancements in fear acquisition and retrieval [42, 43], safety learning [55], and, interestingly, in the extinction learning of socially-cued conditioned fear [54]. A delayed fear generalization [44] also supports prolonged detection of the salient stimulus.

In contrast, these enhancements are not consistently observed in experiments where NPSR1 is transiently inactivated. For instance, studies on inhibitory avoidance [38] and fear extinction learning, particularly those involving NPSR1 inactivation in the amygdala [10] or the VTA [53], have yielded mixed results. Notably, conditioned avoidance behavior is indeed facilitated by blocking NPSR1 in the BNST [67], while the acquisition [36] and extinction [27] of fear conditioning are enhanced when NPSR1 is temporarily inhibited in the BLA. A small positive effect of *Npsr1*-deficiency was found in T-maze discrimination [19], although *Npsr1* KO mice had a poorer performance in novel object, place, or context discrimination [23].

It is important to note that NPS receptor activation produces robust arousal/hyperlocomotion [20, 38, 58], which needs to be taken into account when measuring learning and memory, using paradigms that are less sensitive to spontaneous locomotor activity.

Most studies on the NPS system and memory used aversive stimuli, but an effect on reward-based learning has also been shown. NPS promotes the reinstatement of extinguished drug-seeking responses [52, 60]. This observation may indicate that NPS signaling influences how strongly conditioned stimuli guide behavior under specific contextual conditions, compared to the change in context or the absence of the conditioning stimulus that led to extinction learning. This modulation relates to its interaction with systems that induce drug-seeking reinstatement [28, 34, 49, 51, 52, 65, 72]. The endocannabinoid system has been related to the NPS system in the reinstatement of conditioned place preference. Under stress, the release of NPS activates orexigenic neurons projecting to VTA, which ultimately activate the dopaminergic system, involved in reward-driven behaviors [37, 124]. The interactions between NPS system and other neurotransmitter systems, including serotonin, endocannabinoids, corticotropin-releasing hormone, as well as glutamatergic, GABAergic, and cholinergic systems, emphasize its integrative function in modulating emotional and cognitive processes.

In summary, the reviewed data suggest that the potential involvement of the NPS system in processes related to stimulus relevance during memory formation might depend on the localization of its action in the brain and the nature of the memory task. The varying results from studies on constitutive KO animals or temporary inactivation of the NPS system highlight the different impacts of lifelong dysfunction vs. temporary inactivation on fear memory formation. While current research largely focuses on adult animals, it is crucial to explore differences in NPS system across the lifespan. Future studies encompassing various age groups could enhance our understanding of the neurobiological effects of the NPS system in memory formation.

## Conclusions

Neuropeptide S has been identified as a key bioactive peptide in the mammalian brain, influencing fear, anxiety, wakefulness, reward-seeking, and conditioned learning. Its distribution and functions make the NPS system a good candidate as a learning and memory modulator; however, our knowledge of its mode of action on learning and memory at the molecular, regional, and network levels is limited. This review incorporates 53 studies. The findings indicate that the NPS system influences both long- and short-term forms of memory, and the encoding of aversive, neutral, and appetitive stimuli. In episodic memory, the NPS system enhances object recognition, spatial, and sensory memory, mitigating deficits associated with these learning types. However, results regarding the social aspect of episodic memory present conflicting reports. The NPS system plays a critical role in fear-conditioned learning, facilitating extinction learning and enhancing contextual fear memory generalization. It improves inhibitory avoidance consolidation but reduces the fear-potentiated startle response. In reward-driven conditioning, the NPS system enhances the reinstatement of extinguished associations and cue-induced reinstatement, although it diminishes the place preference response. Finally, in short-term and working memory related to the medial prefrontal cortex, the NPS system helps alleviate short-term memory deficits and increases reversal learning.

Taken together, the evidence reviewed here indicates that the NPS system influences multiple forms of learning and memory. Although the available data do not allow for a definitive conclusion that NPS directly regulates motivation and associated affective states related to memory, the reviewed findings suggest that NPS signaling may preferentially affect learning situations involving emotionally or motivationally relevant stimuli. Rather than acting directly on memory encoding, the NPS system appears to shape when, how, and with what emotional weight the learned information influences behavior. This framework reconciles the apparent heterogeneity of NPS effects across tasks, and supports its relevance for disorders characterized by maladaptive salience attribution, such as anxiety and addiction. Its wide influence on different memory types, and the close interaction with key learning and memory neuromodulators such as dopaminergic and noradrenergic systems, and also some others essential for arousal (e.g., orexinergic), suggest that NPS signaling may influence learning and memory by modulating the perception of the behavioral relevance and long-term value of the learning content.

## Supplementary Information

Supplementary Material 1.

## Data Availability

No datasets were generated or analysed during the current study.

## Abbreviations

5-HT: Serotonin
AA: NPSR1 gene A allele homozygotes
ACh: Acetylcholine
aCSF: Artificial cerebrospinal fluid
AcbSh: Nucleus accumbens shell
Amy: Amygdala
AON: Anterior olfactory nucleus
BLA: Basolateral nucleus of the amygdala
BDNF: Brain-derived neurotrophic factor
BNST: Bed nucleus of the stria terminalis
BSA: Bovine serum albumin
Cb: Endocannabinoid
CeA: Central amygdaloid nucleus
CeL: Centrolateral amygdaloid nucleus
Cg: Cingulate cortex
CORT: Corticosterone
CPP: Conditioned place preference
CREB: cAMP-response element binding protein
CR: Conditioned response
CRF1: Corticotropin-releasing factor receptor 1
CRH: Corticotropin releasing hormone
DA: Dopamine
DCS: D-Cycloserine
DMSO: Dimethyl sulfoxide
EGFP: Enhanced green fluorescent protein
EPM: Elevated plus maze
EPN: Endopiriform nucleus
EtOH: Ethanol
FST: Forced swim test
GABA: Gamma-aminobutyric acid
Glu: Glutamate
Google Sch.: Google Scholar bibliographic database
Hipp: Hippocampus
IA: Inhibitory avoidance
i-BLA: Intra-basolateral amygdala
i-BNST: Intra bed nucleus of the stria terminalis
i-CeA: Intra-central amygdala
i.c.v.: Intracerebroventricular
i-DMH: Intra-dorsomedial hypothalamus
i-EPN: Intra-endopiriform nucleus
IL: Infralimbic cortex
i-LA: Intra-lateral amygdala
LA: Lateral amygdala
i-LH: Intra-lateral hypothalamus
LH: Lateral hypothalamus
i-NAc: Intra-nucleus accumbens
i.p.: Intraperitoneal
i-PeF: Intra-perifornical area of the hypothalamus
i-PVN: Intra-paraventricular nucleus [of the hypothalamus]
i-PVT: Intra-paraventricular nucleus of the thalamus
i.v.: Intravenous
i-VTA: Intra-ventral tegmental area
I107: NPSR1-Ile107 variant
KO: Knock-out
LC: Locus coeruleus
LS: Lateral septum
Med: Medial
m/rHAB: High anxiety-related behavior mouse/rat
m/rLAB: Low anxiety-related behavior mouse/rat
m/rNAB: Normal anxiety-related behavior mouse/rat
mPFC: Medial prefrontal cortex
mRNA: Messenger ribonucleic acid
N/A: Not applicable
NA: Noradrenergic system
NAc: Nucleus accumbens
NCGC: NCGC00185684
NMDAR: N-methyl-D-aspartate receptor
NMDA: N-methyl-D-aspartate
N107: NPSR1-Asn107 variant
NPS: Neuropeptide S
NPSR1: Neuropeptide S receptor 1 [protein]
NPSR1: Neuropeptide S receptor 1 [gene, human]
Npsr1: Neuropeptide S receptor 1 [gene, non-human]
Npsr[1] -/-: Homozygous for Neuropeptide S receptor gene absence
Npsr[1] +/-: Heterozygous for Neuropeptide S receptor gene absence
Npsr[1] +/+: Homozygous for Neuropeptide S receptor gene presence
OFT: Open field test
OxR1(A) /Hcrt(R)1: Hypocretin (orexin) receptor 1
OX/HCRT: Orexin/hypocretin
PA: Passive avoidance
PaS: Parasubiculum
PBS: Phosphate buffered saline
periLC: Peri-coerulean region
Pir: Piriform cortex
PKCδ: Protein kinase C delta
PL: Prelimbic cortex
PrS: Presubiculum
PRISMA: Preferred reporting items for systematic reviews and meta-analyses
PubMed: Database of the United States national library of medicine
PVN: Hypothalamic paraventricular nucleus
REM: Rapid eye movement
s.c.: Subcutaneous
Scopus: Scopus citation and abstract database by Elsevier
STM: Short-term memory
Sub: Subiculum
T+: Carriers of the NPSR1 T+ allele
TST: Tail suspension test
US: Unconditioned stimulus
vs.: Versus
VTA: Ventral tegmental area
WoS: Web of Science platform of academic databases hosted by Clarivate
WM: Working memory
Wks: Weeks old
[+]: Positive effects
[-]: Negative effects
[/]: No effect
