# Supplementary material for: Neuropeptide S modulation of learning and memory: a systematic review
Source: Behav Brain Funct. 2026 May 3;22:17. doi: 10.1186/s12993-026-00336-y (PMC13141304; doi:10.1186/s12993-026-00336-y)
Supplement: Supplementary file 1 — Supplementary Material 1. [file 12993_2026_336_MOESM1_ESM.docx]

**Supplementary Information**

**Supplementary Table S1.** Search strategy for relevant scientific publications databases.

| **Database** | **Boolean search command** | **Number of entries (02.05.2025)** |
| --- | --- | --- |
| PubMed | ((((((((((Neuropeptide S[Title/Abstract]) AND (NPS[Title/Abstract])) AND ((memory[Title/Abstract]) OR (learning[Title/Abstract]) OR (cognition[Title/Abstract]) OR (conditioning[Title/Abstract]) OR (retrieval[Title/Abstract]) OR (acquisition[Title/Abstract]) OR (extinction[Title/Abstract]) OR (appetitive[Title/Abstract]))) NOT (Alzheimer's[Title/Abstract])) NOT (Parkinson's[Title/Abstract])) NOT (schizophrenia[Title/Abstract])) NOT (therapy[Title/Abstract])) NOT (therapeutic[Title/Abstract])) NOT (rehabilitation[Title/Abstract])) NOT (patient[Title/Abstract])) | 53 |
| Google Scholar | "Neuropeptide S" AND "NPS" AND ("memory" OR "learning" OR "cognition" OR "conditioning" OR "retrieval" OR "acquisition" OR "extinction" OR "appetitive") -"Alzheimer's" -"Parkinson's" -"schizophrenia" -"therapy" -"therapeutic" -"rehabilitation" -"patient" | 197 |
| Scopus | TITLE-ABS-KEY ( "neuropeptide s" AND NPS) AND TITLE-ABS-KEY ( memory OR learning OR cognition OR conditioning OR retrieval OR acquisition OR extinction OR appetitive ) AND NOT TITLE-ABS-KEY ( Alzheimer's) AND NOT TITLE-ABS-KEY ( Parkinson's ) AND NOT TITLE-ABS-KEY ( schizophrenia ) AND NOT TITLE-ABS-KEY ( therapy ) AND NOT TITLE-ABS-KEY ( therapeutic ) AND NOT TITLE-ABS-KEY ( rehabilitation ) AND NOT TITLE-ABS-KEY ( patient ) | 72 |
| Web of Science | TS=(((“neuropeptide s”) AND (NPS)) AND ((memory) OR (learning) OR (cognition) OR (conditioning) OR (retrieval) OR (acquisition) OR (extinction) OR (appetitive)) NOT (Alzheimer's) NOT (Parkinson's) NOT (schizophrenia) NOT (therapy) NOT (therapeutic) NOT (rehabilitation) NOT (patient)) | 90 |

**Supplementary Table S2.** Glossary

| **Concept** | **Description** |
| --- | --- |
| *Acquisition* | the attainment by an individual of new behavior, information, or skills or the process by which this occur and the period during which progressive, measurable changes in a response are seen (130) |
| *Appetitive* | pleasant stimuli, such as food, water, or sex (130) |
| *Aversive* | implies avoidance or repugnance, but it may merely indicate opposition or dislike, not necessarily fear (90) |
| *Classical (Pavlovian) fear conditioning* | paradigm in which subjects, typically laboratory rodents, are exposed to a neutral sensory stimulus, such as a light, odor, or tone (the conditioned stimulus, CS) that is contingently paired with an aversive one (the unconditioned stimulus, US), typically a footshock. After several pairings, subjects exhibit defensive responses (the conditioned response, CR) when exposed to the CS alone. |
| *Conditioning* | process by which certain kinds of experience make particular actions more or less likely (130) |
| *Consolidation* | neurobiological process by which a permanent memory is formed following a learning experience (130) |
| *Episodic memory* | recollection of personally experienced events situated within a unique spatial and temporal context |
| *Extinction (learning)* | Learning and memory process in which a behavior acquired through Pavlovian or instrumental (operant) learning decreases in strength when the outcome that had reinforced it is removed (131) |
| *Fear memory* | fundamental form of learning through which animals learn to predict aversive events and react appropriately to threats (90) |
| *Inhibitory avoidance* | instrumental conditioning learning paradigm in which the latency of an individual to enter a conditionally aversive environment is measured; animals learn to refrain from moving into a compartment where they can receive a footshock |
| *Object memory* | type of episodic memory that relies on the natural tendency of mammals to investigate novel or familiar objects in novel locations (132) (133) |
| *Operant (Instrumental) fear conditioning* | subjects can use the CR as an instrument to enhance the probability of receiving a US if it is pleasant (food, water, sex) or to decrease it if the US is unpleasant or dangerous  (90) |
| *Procedural memory* | long-term memory for the skills involved in particular tasks. Procedural memory is demonstrated by skilled performance and is often separate from the ability to verbalize this knowledge (130). |
| *Reinstatement* | relapse effect occurring after extinction learning, where the presentation of the US after extinction can cause the extinguished response to recover to the CS. It can occur both after appetitive or after aversive conditioning (131). |
| *Renewal* | relapse effect after extinction, occurring when the context where the original association took place, is presented (ABA renewal). It has been demonstrated in both fear conditioning (tone-shock conditioning) and appetitive conditioning (tone-food conditioning), taste aversion learning, and forms of human associative learning. ABC renewal occurs when conditioning is happening in context A, extinction in context B, and renewal in a third context (131). |
| *Retrieval* | process of recovering or locating information stored in memory 130) |
| *Reversal* | the switch of a cue-outcome association priorly formed between two or more cues and a particular or no outcome (134) |
| *Semantic memory* | general knowledge and schematic representations of events distilled from lifelong experiences, retrieved independently from their original spatial or temporal context |
| *Spatial memory* | ability to orientate ourselves and navigate the surrounding environment (130) |
| *Working memory* | temporary storage system that allows maintaining and manipulating information to perform complex cognitive tasks, such as comprehension, learning, and reasoning (135). |

**Table S3.** Human studies investigating the role of *NPSR1* gene in memory

| **Authors (year)** | **Population** | **Sampling strategy** | **Sample distribution (age, sex, genotype)** | **Memory type, test (incentive)** | **NPS system measurements (target, test)** | **Other measurements** | ***NPSR1* allele influence on memory: positive (+), negative (-), no effect (/)** | **Other *NPSR1*-related findings** |
| --- | --- | --- | --- | --- | --- | --- | --- | --- |
| Glotzbach-Schoon et al., 2013 | Caucasian descent, healthy | larger sample (N = 497); exclusion: prior diagnosis of DSM-IV axis-I, neurological or somatic disorders, illegal drug and alcohol consumption, smoking, medication, pregnancy, left-handedness, being a psychology student | 23.96, SD = 3.14, years old; 33 M / 60 F | VR contextual fear conditioning, aversive (electric stimulus) | genotyping based on blood sample for polymorphic variants in the gene coding  for *NPSR1*, rs324981 A/T Asn 107Ile | anxiety traits questionnaires: trait version of STAI, ASI, BIS-BAS, life stress history self-report questionnaire;  genotyping based on blood sample for 5HTTLPR gene polymorphism | (+) acquisition and extinction of fear conditioning in participants carrying both risk alleles (S+/T+); (+) fear memory consolidation in carriers of one risk allele (S+/AA, T+/LL) | (+) explicit anxiety conditioned responses (self-rated) in AA carriers; (+) implicit responses (startle reaction) in T+ carriers; (-) a negative correlation between the amount of stressful life events and fear conditioning in T+ carriers |
| Guhn et al., 2015 | Caucasian volunteers; 12.91 +/- 0.5 years of education | screened for: current mental health, and right-handedness | 25.36 +/- 4.8 years old; 33 M / 33 F | working memory, emotional n-back task, aversive (emotion-evoking pictures) | genotyping for *NPSR1* s324981 A/T variant (AA homozygotes and T allele carriers) | state and trait measurements: STAI, ASI; current mental health: INI; fNIRS; skin conductance; right handedness: Edinburgh Handedness Inventory | (/) *NPSR1* genotype differences in accuracy, reaction times, and skin conductance; in the high working memory load condition: T+ carriers signal (+) in response to negative pictures, and signal (-) in response to positive pictures; AA homozygotes signal (-) to positive and (+) to negative pictures, in the mPFC and left dlPFC as measured with fNIRS | in T+ carriers, a high anxiety index correlated with (-) signal in mPFC and (+) activation in left dlPFC |
| Raczka et al., 2010 | healthy male, right-handed | preselected for polymorphisms in the dopamine transporter and the catechol-o- methyltransferase genes | 28 *NPSR1* (AA), 23–40 years old /  38 *NPSR1* (TT), 19–42 years old; 66 M / 0 F | cued fear conditioning, aversive (electric stimulus) | homozygous  *NPSR1* AA allele (AA), and one or two *NPSR1* T  alleles (T+) | genotyping for BDNF and serotonin transporter SLC6A4 | (+) acquisition in *NPSR1* T allele carriers on self-reported fear reactions to CSs; (/) acquisition in any genotype as seen in skin conductance responses to CS | (+) CS-evoked activation in the rostral dorsomedial prefrontal cortex in T carriers correlating with increased self-rated fear level |

**Legend:** (+): positive effect; (-) negative effect; (/): no effect; ASI = Anxiety-Sensitivity-Index; BDNF = brain-derived neurotrophic factor; CS = conditioned stimulus; BIS-BAS = Behavioral Inhibition System and Behavioral Approach System; dlPFC = dorsolateral prefrontal cortex; DSM-IV = Diagnostic and Statistical Manual of Mental Disorders, fourth edition; F = female; fNIRS = Functional near infrared spectroscopy; INI = International Neuropsychiatric Interview; mPFC = medial prefrontal cortex; NPSR1 = Neuropeptide S receptor 1; M = male; SD = standard deviation; STAI = State-Trait-Anxiety-Inventory; VR = virtual reality

**Table S4a** Studies investigating the role of NPS system in memory using genetically modified rodent models

| **Authors (year)** | **Animal model (species, strain)** | **Genetic intervention (target genes, groups)** | **Sample distribution (age, weight, sex)** | **Memory type, test (incentive)** | **NPS(R) deficiency effects on memory** | **NPS(R) facilitation effects on memory** | **Other NPS-related findings** |
| --- | --- | --- | --- | --- | --- | --- | --- |
| Bengoetxea et al., 2021 | Mouse, C57BL/6 | constitutive knock-in, Tacem3778(I107) Tac (*Npsr1-*N107 humanized) and *Npsr1*-I107 (I107) | 121 (59 M, 63 F), 9–13 wks | conditioning, Pavlovian, auditory-cued, aversive (footshock) | (+) extinction of conditioned fear (sex-specific) | N/A | (-) *Npsr1* signaling and feedforward inhibition in the BLA synaptic circuits in the human-specific hypofunctional N107 genotype |
| Bicakci et al., 2021 | Mouse, C57BL/6J | constitutive knock-out, *Npsr1*-deficient mice B6.129S6/SvEvTac-Npsr1tm1Bhk | 40 WT and 42 *Npsr1*−/+and homozygous *Npsr1*−/− M and F, 8–12 wks | spatial memory, T-maze discrimination and reversal learning task, appetitive (sucrose pellets) | (+) T-maze discrimination performance on the last day of acquisition in *Npsr1*−/− mice compared to *Npsr1*+/+ mice; (+) proportion of learners in *Npsr1* −/− mice; (/) T-maze reversal learning difference between genotypes; (/) learning strategy (allocentric vs egocentric) difference between genotypes | See Table S4b | See Table S4b |
| Duangdao et al., 2009 | Mouse, 129S6/SvEvTac | constitutive knock-out, *Npsr1* KO and WT | approx. 375 M (total from reported groups), 8–15 wks | prepulse inhibition of the startle response, aversive (tone) | (/) influence on startle habituation and pre-pulse inhibition by *Npsr1*-deficiency | N/A | (-) locomotion in the OFT; (-) late peak wheel running activity; (+) anxiety levels; (+) motor performance skills; (/) forced-swimming |
| Fendt et al, 2011 | Mouse, C57BL/6J; 129S | constitutive KO, *Npsr1* +/+, *Npsr1* +/-, *Npsr1* -/- | approx. 206 M (total from reported groups),  12–25 wks | conditioning, Pavlovian, auditory-cued, aversive (footshock); prepulse inhibition of the acoustic startle response, aversive (tone) | (/) cued fear in *Npsr1* -/- mice; modest (+) contextual fear (1-min test) in *Npsr1* -/- mice; (/) genotype effects in extinction of contextual fear; (/) effects of *Npsr1*-deficiency on prepulse inhibition | N/A | See Table S4b  (/) locomotor activity in *Npsr1*-deficient mice induced by NPS injection; (+) locomotor activity in *Npsr1*-deficient mice induced by cocaine; in *Npsr1* -/+ and *Npsr1* -/-: (-) locomotion during the active phase with lights-off; (/) locomotion during the passive phase with lights-on; (/) genotype differences in open field activity; weak (+) anxiogenic-like effect in the second exposure to the elevated plus maze; robust (-) startle response magnitude in *Npsr1* heterozygous and homozygous; (/) genotype effects on haloperidol modulation of startle amplitude |
| Garau et al., 2022 | Mouse, C57Bl/6J; 129S6/SvEvTac | constitutive KO, *Npsr1* KO; NPS precursor | 245 M, 8–12 wks | conditioning, Pavlovian auditory-cued, aversive (footshock); conditioned place preference, appetitive (morphine); spatial memory, Morris Water maze, appetitive (escape); object memory, novel object recognition, appetitive (novelty) | (+) acquisition of conditioned fear in *Npsr1* knockout (KO) at footshock intensities < 0.8 mA; (+) recall of conditioned freezing in *Npsr1* KO at shock intensities < 1.0 mA; (+) acquisition and recall of conditioned fear in NPS precursor KO at shock intensities 0.4–0.8 mA; (-) extinction learning in both *Npsr1* and *NPS* precursor KO at shock intensities of 0.8 mA; (/) renewal of fear conditioning in either of the *Npsr1*-deficient genotype; (/) acquisition, consolidation or reversal learning in a water maze spatial memory task for either of the *Npsr1*-deficient genotype; (/) novel object recognition in both *Npsr1*-deficient genotypes | N/A | See Table S4b |
| Germer et al., 2019 | Mouse, C57BL/6J | *Npsr1* +/d (*Npsr1*-haplodeficiency) and *Npsr1* d/d (*Npsr1*-deficiency) or *Npsr1* +/d | 95 F, 79 M: 56 +/+, 64 +/d, 54 d/d, 8–12 wks | conditioning, contextual fear conditioning, aversive (footshock) | (/) difference between *Npsr1*-deficient and WT littermates or between sexes in contextual fear conditioning acquisition and retention; (-) trend in reactivity to the electric stimulus in *Npsr1* +/d and *Npsr1* d/d ; (+) freezing in *Npsr1* +/d and *Npsr1* d/d mice compared to *Npsr1* +/+ littermates in the retention test (3 min duration); (-) generalization of the contextual fear memory after an incubation time of one week but not at 4 weeks in *Npsr1* +/d and *Npsr1* d/d | NA | (+) freezing generally expressed in F than in M mice (irrespective of context); (+) startle magnitude and light-dark box anxiety-like behaviors in M than in F; (-) startle magnitude in *Npsr1* +/d but not *Npsr1* d/d mice after 4 weeks; (+) anxiety-like behaviors in the light-dark box in *Npsr1*-deficient mice; (/) difference between genotypes in corticosterone plasma levels after one trial contextual conditioning; (+) CORT plasma levels increase after 4 weeks incubation time from contextual fear conditioning in *Npsr1* +/d and *Npsr1* d/d compared to *Npsr1* +/+ mice; (+) CORT plasma level in *Npsr1* d/d than in *Npsr1* +/+ mice at 1 week post-conditioning |
| Jüngling et al., 2015 | Mouse, C57Bl/6J | EGFP marker in NPS neurons, NPS-EGFP line E16 | approx. 24 mice (based on reported groups) of both sexes with distribution not specified, age not specified | conditioning, Pavlovian, auditory-cued (paired and unpaired), aversive (footshock) | NA | (+) fear memory retrieval relies on dynorphin and somatostatin-expressing neurons in the centrolateral amygdala that send GABAergic projections to the NPS neurons in the locus coeruleus | (+) NPS neurons in the LC and peri-LC are innervated by specific fear-on neurons in the CeL Amy lacking PKCδ and expressing prodynorphin and somatostatin; (-) dynorphin and somatostatin inhibit the NPS neurons via postsynaptic receptors stimulation |
| Kolodziejczyk et al., 2020 | Mouse, C57Bl/6J | constitutive KO, *Npsr1*-  deficient (heterozygous, homozygous, and WT) | approx. 226 M and F (total from reported groups), sex distribution not specified, 8–16 wks | episodic memory, social novelty test, appetitive (novelty); conditioning, social fear conditioning, aversive (footshock) | (-) social novelty recognition in heterozygous *Npsr1*-deficient females; (/) social novelty recognition in *Npsr1*-/- of both sexes and in male *Npsr1* -/+; (/) acquisition and expression of conditioned social fear irrespective of *Npsr1* genotype; (-) extinction of conditioned social fear in *Npsr1* -/+; (+) extinction of conditioned social fear in homozygous *Npsr1* -/- mice | NA | (-) *Npsr1* +/- and M mice locomotor activity |
| Kreutzmann et al., 2020 | Mouse, C57Bl/6J | homozygous (*Npsr*−/−), heterozygous (*Npsr* +/-), & WT ( *Npsr* +/+) | 134 F and 125 M, 168 WT and 91 *Npsr1*-deficient mice, 8–12 wks | conditioning, safety learning, aversive (footshock) | *Npsr1* −/− without pre-exposure to stress: (+) safety learning in M; (/) safety learning in F; *Npsr1* −/− pre-exposed to electric stimuli stress: (-) lack of pre-stress learning enhancement without affecting baseline safety learning in M; (/) safety learning in either pre-exposure situations in *Npsr1*−/− F | N/A | (/) safety learning (freezing to a novel context) performance influenced by lack of stress or pre-exposure to immobilization stress in WT mice; (+) safety learning with pre-exposure to stress induced by electric stimuli in M but not F WT and *Npsr1* +/+ mice |
| Liu et al., 2017 | Mouse, C57BL/6NTac; C57BL/6J | constitutive, (*Npsr1* precursor, mRNA, KO), WT, (*Nps*+/+), *Nps* knockout (KO, *Nps*-/-), and NPS heterozygous (*Nps*+/-) | 224 M, 8–15 wks | conditioning, Pavlovian, auditory-cued and inhibitory avoidance, aversive (footshock) | (-) long-term memory in *Nps*-/- mice in the inhibitory avoidance paradigm | N/A | (-) locomotion in the OFT; (-) arousal in the hole board test; mild (-) in anxiety-like behaviors |
| Okamura et al., 2011 | Mouse, C57Bl/6; 129S6/SvEvTac | constitutive, *Npsr1* heterozygous and knock-out, WT | approx. 115 (total from reported groups) M, 8–14 wks | episodic memory, novel object/ place/context recognition, appetitive (novelty); inhibitory avoidance, aversive (footshock) | (-) 48-h retention latencies in the inhibitory avoidance test of *Npsr1* KO compared with their heterozygous and wild-type littermates; (-) object memory, and novel place and novel context recognition of *Npsr1* KO | N/A | See Table S4b |
| Rappeneau et al., 2025 | Mouse, C57BL/6NTacem3778  (I107N)Tac (*NPSR1*-I107N) | generated through CRISPR/  Cas9-mediated knock-in, point mutation in exon 4 of mouse *Npsr1* | 51 M and 37 F, 43–45 per genotype, 8–12 wks | spatial working memory, spontaneous alternation test, neutral; working memory and reversal learning, attentional set shifting task, appetitive (food) | N/A | *NPSR1*-N107 *vs. NPSR1*-I107 mice: (/) spontaneous alternation; (/) social novelty recognition; (+) reversal learning in *NPSR1*-N107 line | *NPSR1*-N107 *vs. NPSR1*-I107 mice:  (+) anxiety level in *NPSR1*-N107 mice; (/) arousal; (/) exploratory behavior; (/) hormonal stress response |
| Ruzza et al., 2012 | Mouse, CD-1 | constitutive KO, *Npsr1* (+/+) and *Npsr1* (-/-) | approx. 325 M (total from reported groups), 8–10 wks, 28–35 g | episodic memory, novel object recognition, appetitive (novelty) | (/) genotype effect on novel object recognition (tested at 3 h after the learning trial) | N/A | in NPS-injected WT mice: (+) locomotor activity; (/) exploratory activity difference between genotypes; (/) genotype differences in the righting reflex, sleep time, nociceptive behavior (in the formalin test) or anxiety-like behaviors in the elevated plus maze; (-) rearing activity in the OFT in the *Npsr1*-deficient mice; (/) 1 nmol NPS on rearing and anxiety-like behavior in the OFT in *Npsr1*-deficient compared to WT mice |
| Smith et al., 2014 | Mouse, C57BL6/N and retired Hsd: ICR (CD1) breeders, WT | N/A (measured *Nps* mRNA following memory-related activity) | 65 M (50 C57BL6/N of ~ 23 g, 15 CD1 of ~ 53 g), 8 wks | conditioning, Stress Alternative Model (social aggression cued auditory) and Pavlovian (footshock cued auditory), aversive (footshock or social aggressor) | N/A | (+) expression level of *Nps* mRNA in submissive mice (following conditioning) | (+) *Nps* mRNA expression and (-) BDNF mRNA expression in the CeA; (+) corticosterone plasma level in all socially interacting mice |
| Xing et al., 2019 | mouse, C57BL/6J | *Npsr1*-Y206H knock-in, mutation using CRISPR-Cas9, wild-type (WT; *Npsr1*+/+) and mutant (*Npsr1*+/m) | 283 M (total from reported groups; behavioral tests only), minimum 8 wks | conditioning, contextual fear conditioning, aversive (footshock) | N/A | (+) resilience to sleep loss of contextual memory in *Npsr1*-Y206H mice | (+) mobile time and traveled distance, but in less episodes than WT; (-) time sleeping spent in *Npsr1*-Y206H mice; (+) sleep pressure sustained in *Npsr1*-Y206H mice; (/) recovery sleep after sleep deprivation; (+) phospho–cAMP response element–binding protein in the brain; (+) neuronal sensitivity to NPS |
| Zhu et al., 2010 | Mouse,129Sv/J × C57BL/6 | *Npsr1* deficient | total number not specified, M and F, 8–10 wks | episodic and spatial memory, Morris water maze task, appetitive (escape); prepulse inhibition of the acoustic startle response, aversive (tone) | (/) differences between genotypes in spatial memory acquisition and retrieval;  (-) differences in prepulse inhibition of the startle response in *Npsr1*-deficient male mice | N/A | in *Npsr1*-deficient mice: (/) basal anxiety levels and locomotion (in EPM, marble burying, LDB, and OFT, including after (+)- methamphetamine challenge); (+) depression-like behavior in FST but not TST; (-) acoustic startle response; (/) plasma corticosterone differences between WT and *Npsr1*-deficient mice (after locomotion or forced swim); (+) dose-response effects on plasma corticosterone 40 min after NPS i.c.v. injection in WT mice; (+) vertical and horizontal activity and anxiolytic effects in WT but not *Npsr1*-deficient mice following NPS injection |

**Legend:** (+) =positive effect; (-) = negative effect; (/) = no effect; Amy = amygdala; CeL = centrolateral; CORT = corticosterone; EGFP = enhanced green fluorescent protein; EPM = elevated plus maze; F = female; FST = forced swim test; GABA = Gamma-aminobutyric acid; ; i.c.v. = intracerebroventricular; KO = knock-out; (peri) LC = (peri) locus coeruleus; LDB = light-dark box; M = male; N/A = not applicable; *Npsr(1)* -/- = homozygous; *Npsr(1*) +/- = heterozygous; *Npsr(1)* +/+ = no *Npsr(1)* genetic deficiency; OFT = open field test; PKCδ = protein kinase C delta; wks = weeks (old); WT = wild type; TST = tail suspension test

**Table S4b** Studies investigating the role of NPS system in memory using pharmacological interventions in rodents

| **Authors (year)** | **Animal model (species, strain, genotype)** | **Pharmacological intervention (drugs, concentration, administration)** | **Sample distribution (age, weight, sex)** | **Memory (type / test / incentive)** | **NPS(R) deficiency effects on memory** | **NPS(R) facilitation effects on memory** | **Other NPS-related findings** |
| --- | --- | --- | --- | --- | --- | --- | --- |
| Bengoetxea et al., 2021 | Mouse, C57BL/6NTacem3778(I107N)Tac (*Npsr1*-N107 humanized) and *Npsr1*-I107 | SHA68 (NPSR1 antagonist) − 300 μl, bilaterally i-BLA | approx. 121 M (total from reported groups), 9–13 wks | conditioning, Pavlovian, aversive (footshock) | (+) extinction of conditioned fear | N/A | See Table S4a |
| Bicakci et al., 2021 | Mouse, C57BL/6, WT | NPS (1 mM) − 10 μl, nasal | 40 M, 8–12 wks | spatial memory, T-maze, appetitive (sucrose pellet) | N/A | (/) acquisition; (/) nasal NPS influence on T-maze discrimination learning in M WT mice; (+) reversal | (/) effect on locomotion |
| Cannella et al., 2009 | Rat, Wistar, WT | SB334867 (non-peptide OxR1/Hcrt-r1 antagonist) − 10 mg/kg, i.p.; NPS (1, 2, 4 nmol) − 1 ml, i.c.v. or i-LH; alcohol (10%), orally | approx. 59 M (total from reported groups), 175–225 g | conditioning, reinstatement of alcohol-seeking behavior, appetitive (alcohol) | N/A | i.c.v. NPS: (/) acquisition; (+) retrieval; i-LH NPS: (+) retrieval; i-LH NPS & ip SB334867: (/) retrieval | i-LH NPS: potentially (+) hyperarousal (as seen in inactive lever pressing) |
| Cannella et al., 2016 | Rat, Wistar & Marchigian Sardinian alcohol-preferring (msP), WT | NPS (0.1, 0.5, 1.0, 2.0 nmol1) − 1 μl, i.c.v. | approx. 116 M (total from reported groups), 7–9 wks, 175–225 g | conditioning, cued operant, appetitive (alcohol) | N/A | (-) acquisition of self-administration; (/) reinstatement in msP rats; (+) reinstatement in Wistar rats | (-) anxiolytic effects in msP rats; (/) effect in Wistar rats |
| Cao et al., 2011 | Rat, Wistar, WT | NPS − 0.34–34 pmol per self-admin infusion & 10, 100 or 1000 pmol/injection, i.c.v.; R(+)-SCH 23390 (D1 receptor antagonist) − 0.025 mg/kg, i.p.; SB 334867 (OxR1/Hcrt-r1 antagonist) − 20 mg/kg, i.p. | 98 M, 280–350 g | conditioning, operant (NPS self-administration assisted by visual cues and place conditioning), (putatively) appetitive (NPS) | N/A | i.c.v. NPS: (+) retrieval; NPS + SCH or SB: (-) retrieval; NPS on place conditioning: (+) high dose, (-) medium dose, (/) low dose | (/) effect on general arousal or hyperactivity; high and medium i.c.v. NPS doses: (+) locomotion and rearing (acute only, no sensitization effect) |
| Chauveau et al., 2012 | Mouse, C57BL/6, WT | NPS (10 μM) − 0.5 μl bilaterally i-LA; SHA68 (NPSR antagonist) − 10 μM, 0.5 μl bilaterally i-LA | approx. 75 mice (total from reported groups), sex not specified, 8–12 wks | conditioning, Pavlovian, aversive (footshock) | i-LA SHA68 in stress pre-exposed condition: (+) acquisition | i-LA NPS in stress pre-exposed condition: (-) acquisition; (+) extinction | (+) NPS in LA anxiolytic effects both in conditioned fear behavior and in excitability of LA neurons upon fear extinction |
| Chou et al., 2021 | Mouse, C57BL/6, WT | cocaine hydrochloride − 20 mg/kg, i.p.; NPS (1 nmol) − 1 μl i.c.v. or 0.5 μl, bilateral i-VTA; SHA68 (NPSR antagonist) − 50 mg/kg, i.p.; SB-334867 (OX1R antagonist) − 15 mg/kg, i.p. or 15 nmol, i-VTA.; AM 251 (CB1 receptor antagonist) − 1.1 mg/kg i.p. or 30 nmol, i-VTA | approx. 245 M (total from reported groups), 8–12 wks | conditioning, place preference, appetitive (cocaine) | N/A | (+) reinstatement of extinguished conditioned place preference by i.c.v. NPS | (+) NPS facilitation of the number of c-Fos-containing orexin neurons in the LH and of orexin-A level in the VTA |
| Clark et al., 2017 | Mouse, C57BL/6, WT | RTI-118 (NPSR antagonist) − 50 mg/kg, i.p.; NPS − 2 μl, i.c.v.; Compound 4 (C4, biased NPSR1 agonist) − 2 μl, i.c.v. | 347 M (263 with proper cannula placement), 8–12 wks | conditioning, inhibitory avoidance, aversive (footshock) | N/A | (+) consolidation (NPS or C4) | (+) locomotion and anxiety (C4 or NPS) |
| Costa et al., 2024 | Mouse, C57BL/6NCr, WT | NPS (0.5 mM, dissolved in artificial cerebrospinal fluid with 0.1% BSA) − 0.01, 0.1 and 1 nmol, i.c.v. | 227 F, 8–9 wks | associative memory, inhibitory avoidance, aversive (footshock) | N/A | (+) consolidation of IA in F mice with low estrogen injected with 0.1 and 1 nmol NPS | (+) locomotion (independent on estrous cycle); (-) anxiety (only in high estrogen stages); (+) Acclimatized startle during high estrous cycle; Reduction of startle magnitude with previous 1 nmol NPS treatment. |
| Enquist et al., 2012 | Mouse, C57BL/6, WT | NPS (3 nmol) − 2 μl, i.c.v. or 1 nmol in 0.3 μl bilaterally i-BLA; EtOH (20%), orally; morphine − 10 mg/kg, subcutaneously | approx. 382 M (total from reported groups), 8 wks | conditioning, place preference and avoidance, aversive (illuminated open space) and appetitive (alcohol or morphine) | N/A | (/) retrieval of conditioned place preference by i.c.v. NPS | (+) anxiety and depression; (+) locomotion; (+) GABA inhibitory postsynaptic current in the BLA after EtOH + NPS consumption |
| Fendt et al, 2010 | Mouse, DBA1/J, WT | NPS (0.01; 0.1, 1 nmol/side), bilaterally i-BLA | 60 M, 8–12 wks | episodic/ associative, fear-potentiated startle response, aversive (footshock, light, sound) | N/A | (-) i-BLA NPS (1 nmol) blocks the expression of conditioned fear independent of increased locomotion | (+) distance traveled following i-BLA NPS (1 nmol) |
| Fendt et al, 2011 | Mouse, C57BL/6, *Npsr1* +/+, *Npsr1*+/- & *Npsr1*-/- | NPS (1 nmol) − 0.3 μl bilaterally, i.c.v.; cocaine − 15 mg/kg, i.p.; haloperidol − 0.3 mg/kg, i.p. | approx. 225 M (total from reported groups), 12–25 wks | prepulse inhibition of the acoustic startle response, aversive (tone) | (/) effects of *Npsr1*-deficiency on haloperidol-facilitated prepulse inhibition (in either *Npsr1* +/+, *Npsr1* +/- & *Npsr1* -/- mice) | N/A | (+) locomotion in WT by i.c.v. NPS or cocaine; (/) locomotion in *Npsr1*-/- by i.c.v. NPS or cocaine |
| Garau et al., 2022 | Mouse, C57Bl/6J, WT and *Npsr1*KO mice (129S6/SvEvTac  background); NPS precursor mice | NPS (1 pmol) − 0.2 μl, i-PVT or intra-3rd ventricle; [D-Cys(tBut)5]-NPS (NPSR1 antagonist, 100 pmol) − 0.2 μl, i-PVT or intra-3rd ventricle; morphine sulphate − 100 μl, i.p. | approx. 245 M (total from reported groups), 8–12 wks | conditioning, auditory, aversive (footshock);  episodic and spatial memory, Morris water maze; appetitive (escape);  episodic, novel object recognition, appetitive (novelty);  conditioning, place preference, appetitive (morphine) | i-PVT [D-Cys(tBut)5]-NPS in WT: (-) consolidation; (/) extinction | (+) extinction and retrieval of conditioning after i-PVT NPS in NPS precursor KO mice; (/) acquisition of conditioning after intra-3rd ventricle NPS injection in NPS precursor KO mice; (+) consolidation of episodic memory after i-PVT of NPS in NPS precursor KO mice; (/) extinction in NPS precursor KO mice by 1 pmol NPS intra-3rd ventricle | (+) c-Fos expression after NPS i-PVT in AcbSh, LS, IL and PL, and Med Amy |
| Han et al., 2009 | Mouse, Kunming, WT | NPS (0.1, 0.3, 1 nmol) − 2 μl, i.c.v.; MK801 (NMDAR antagonist) − 0.1 mg/kg, i.p. | approx. 94 M (total from reported groups), 18–22 g | episodic and spatial memory, Morris water maze, appetitive (escape) | N/A | (+) acquisition of spatial learning by 1 nmol i.c.v. NPS; (+) NPS mitigated MK801 spatial learning impairments | (/) swimming speed by i.c.v. NPS (although p = 0.07) |
| Han et al., 2014 | Mouse, Kunming (Swiss), WT | NPS (1nmol) - i.c.v. or i-BLA (0.5 nmol/side); Propranolol 2mg/kg, 10 ml/kg, i.p. or bilaterally i-BLA (0.5 μg/side) with NPS (0.5 nmol/side) | approx. 105 M (total from reported groups), 20–24 g | episodic, novel object recognition, appetitive (novelty) | N/A | (+) consolidation of object memory by either i.c.v. or i-BLA NPS; (-) propranolol blocks enhancing effect of NPS | N/A |
| Huang et al, 2023 | Rat, Sprague Dawley, WT | NPS (1 nmol) − 2 μl, i.c.v.; RTI-263 (NPSR1 biased agonist, 0–10 nmol) − 2 μl, i.c.v.; RTI-118 (NPSR1 antagonist, 10 nmol) − 2 μl, i.c.v.; Cocaine − 1 mg/ml, i.v. | approx. 215 M and F rats (total from reported groups), sex distribution not specified, weight: 301–325 g and 250–275 g M, and 190–200 g F | operant conditioning, cue-induced sucrose seeking, appetitive (sucrose, cocaine) | (-) RTI-118 effect on operant conditioning (cocaine-seeking behavior) | (+) seeking behavior in reinstatement by NPS; (-) responding in cue-induced reinstatement of cocaine seeking by RTI-263 | RTI-263: (/) locomotion effects; (-) antagonizes locomotor effects of NPS; (-) anxiety-like behavior in the light-dark box; (/) arousal effects; (/) effects on palatable food intake; (-) NPS-mediated intake decrease; (/) feeding during fasting; (/) cue-induced reinstatement of sucrose seeking |
| Jüngling et al., 2008 | Mouse, C57BL/6, WT | SHA68 (10uM) − 0.5 μl each side, i-LA/BLA; NPS (10uM), 0.5 μl each side, i-LA/BLA | approx. 162 mice (total from reported groups), sex not specified, 8–12 wks | conditioning, Pavlovian, aversive (footshock) | (-) extinction of conditioned fear by i-LA/BLA of SHA68 | (+) extinction of conditioned fear by i-LA/BLA of NPS | (-) anxiety by i-LA/BLA of NPS; (-) SHA68 on anxiolytic effect on NPS; (+) locomotion by i-LA/BLA of NPS; (-) SHA68 on hyperlocomotion effect of NPS; (+) synaptic transmission at Glu synapses on paracapsular GABAergic cells by NPS, through receptors located in presynaptic principal neurons |
| Kallupi et al, 2010 | Rat, Long Evans, WT | Cocaine HCl (0.25 mg/0.1 mL), i.v.; SB-334867 (in 10% 2-hydroxypropyl -β-cyclodextrin and 1% DMSO), i.p.; SHA68 - NPS antagonist (in 10% Cremophor EL, 4% DMSO, and 86% of distilled water), i.p.; NPS (in saline), i.c.v., i-LH, -PeF, -CeA, -DMH; [D-Cys(tBut)5]NPS - NPS antagonist (in saline), 1 μl i.c.v., 0.3 ul/site i-LH, -PeF, -CeA and -DMH | approx. 94 M (total from reported groups), age or weight not specified | operant conditioning, self-administration and cue-induced reinstatement, appetitive (cocaine) | (/) reinforcement; (-) conditioned reinstatement (SHA68 NPS antagonist − 30 and 60 mg/kg) | (/) reinforcement; (+) conditioned reinstatement (2 nmol NPS) | (/) Cocaine self-administration by NPSR1 activation or blockade; (+) NPS intra-LH on relapse to cocaine seeking elicited by contextual cues, concomitant with LH Hcrt-1/Ox-A neurons activation |
| Kallupi et al, 2013 | Rat, Wistar, WT | Cocaine HCl (0.25 mg/kg/inf), i.v.; NPSR1- QA1 (1 mg/ml) - NPS antagonist, i.p.;  [D-Cys(tBut)5]NPS - NPS antagonist (1 μl i.c.v., 0.3 μl/side i-LH, i-PeF, and i-CeA) | 70 M, 250–300 g | operant conditioning and reinforcement, appetitive (cocaine or food) | i.p. NPSR1- QA1: (-) reinstatement for cocaine-seeking; (/) retrieval for cocaine-seeking; high dose only: (-) retrieval of food-seeking; i-LH and i-PeF but not i-CeA or i.c.v. [D-Cys(tBu)5]NPS: (-) relapse of cocaine self-administration. | N/A | N/A |
| Kawade et al, 2022 | Rat, Wistar, WT | NPS (0.01, 0.1, 0.5 nM), SHA-68 (10 μM), aCSF or 10% Cremophor-PBS - i-VTA, 0.25 μl/side | 90 M, adult (age not specified) | conditioning, Pavlovian, aversive (footshock) | (-) fear extinction by SHA-68 i-VTA | (+) fear extinction by NPS i-VTA | (+) influx of dopamine in the AcbSh and c-Fos immunoreactive cells in AccSh by NPS treatment |
| Kolodziejczyk and Fendt, 2020 | Mouse, C57BL/6, WT, *Npsr1*−/−, *Npsr1*+/− | 2.5 mg/kg, or 5 mg/kg of CORT, i.p. (10 ml/kg) | approx. 173 (total from reported groups): 90 M and 83 F, 8–12 wks | conditioning, Pavlovian, aversive (auditory and footshock, the unpaired cue-shock protocol) | (-) 5 mg/kg CORT-treated *Npsr1*−/− mice fear memory consolidation (at 24–48 h and 1 month); (-) generalized contextual fear memory; (/) fear inhibition in *Npsr1*+/− and *Npsr1*−/− mice. | N/A | (/) anxiety-like behavior in the light–dark box test; (-) startle responses after the incubation time in 5 mg/kg CORT-treated *Npsr1*−/− mice; (-) gain in body weight in the same group |
| Li et al., 2009 | Mouse, Kunming, WT | saline or morphine, 0.3, 1, 3, and 6 nmol, i.c.v.; NPS (3 μl), 0.1, 0.3, 1, 3, 6 and10 nmol, i.c.v. | approx. 403 M (total from reported groups), 18–22 g | conditioning, place preference, appetitive (morphine) | N/A | (/) NPS (0.3, 1, 3, 10 nmol) on place preference or aversion; (-) NPS (1 and 3 nmol) co-injected with 3 nmol morphine, or NPS (6 and 10 nmol) co-injected with 6 nmol morphine, on CPP acquisition; (-) NPS (0.1, 1 and 10 nmol) injected 15 min prior acquisition on CPP expression induced by 6 nmol morphine | NPS: (+) locomotor activity; (/) morphine-induced hyperlocomotion |
| Li et al., 2022 | Mouse, C57BL/6, Thy1-YFP-H (transgenic mice expressing YFP in cortical layer V pyramidal cells) | NPS 1nmol, dissolved in saline, i.c.v.; SHA68, dissolved in saline 5% DMSO and 10% Cremophor EL, i.p. | approx. 160 M and F (total from reported groups), sex distribution not specified, 4–5 wks | motor memory (skill), rotarod test, neutral incentive | N/A | (+) NPS (1nmol, i.c.v.) treatment post-acquisition on motor memory performance (consolidation) | NPS: (+) survival of newly formed spines induced by motor training and strength of persistent new spines; (-) elimination of existing spines 0–8 h after administration;  (+) Ca^2+^ activity of apical dendrites and dendritic spines of layer V pyramidal neurons in the primary motor cortex during running |
| Li et al.,2025 | Rat, msP | NPS 0.5 nmol,1 and 2nmol i.c.v. | 48 F and 39 M, age not specified | Conditioning, Pavlovian, aversive (footshock), Operant self-administration of alcohol (appetitive), extinction, and reinstatement | N/A | (+) extinction of fear memory in F alcohol preferring rats (-) alcohol self-administration (/) yohimbine induced alcohol seeking reinstatement | NPS 0.5 and 1 nmol (+) locomotor activity, especially in M; 1nmol (+) time in the open arms of Elevated Plus Maze in M rats (alleviates anxiety). |
| Lukas & Neumann, 2012 | Rat, H6164, WT | sterile Ringer solution (5 μl) or NPS (1 nmol/5 μl; i.e. 2 ug/5 μl) i.c.v.; sterile Ringer solution (2 × 10 μl) or NPS (0.4, 4.0, or 40 nmol/2 × 10 μl; i.e. 0.8, 8.0, or 80 μg/2 × 10 μl, intra-nasal; sterile Ringer solution (500 μl) or NPS (similar doses as for intra-nasal but in 500 μl) - s.c.; NPS-A (antagonist, 10 nmol/5 μl; i.e. 30 μg/5 μl) - i.c.v. | approx. 310 M (total from reported groups), 230–300 g | episodic memory, object and social discrimination, appetitive (novelty) | N/A | (+) NPS (1nmol, i.c.v., applied immediately after acquisition) on object discrimination (consolidation); (/) NPS (1nmol, i.c.v., applied 30 min prior testing) on social memory and social preference or avoidance (retrieval); (+) NPS (4-40nmol, applied prior acquisition, nasal) on object discrimination (acquisition) | (-) NPS (4–40 nmol, nasal) on anxiety behaviors in the EPM; (/) NPS (4–40 nmol, s.c.) on anxiety behaviors in the EPM; (/) NPS-A (10 nmol, i.c.v, 30 min prior to acquisition) on social preference |
| Meis et al., 2008 | Mouse, C57BL/6BomTac inbred line | NPS (0.1 nmole; 0.01 nmole), i-EPN | 38 M,10–14 wks | conditioning and fear generalization, Pavlovian, aversive (auditory and footshock) | N/A | (-) NPS (0.1 nmol) on contextually conditioned fear behavior (freezing and risk assessment); (/) NPS on auditory cued fear memory; (/) NPS on anxiety generalization to a neutral tone | i-EPN NPS: (+) GABA-mediated synaptic activity in BLA projection neurons; (+) Glu synaptic activity in BLA interneurons and projection neurons; (+) activation of an inward current in 20% of EPN neurons and Glu excitation in the EPN; (/) anxiety-like or exploratory behavior in the EPM |
| Okamura et al., 2011 | Mouse, C57BL6, *Npsr1*-KO (129S6 background) | NPS (2 μl), i.c.v.; SHA68 (100 μl), i.p.; Propranolol (100 μl), i.p. | approx. 434 M (total from reported groups), 8–14 wks | episodic memory, object memory, inhibitory avoidance, appetitive (novelty) and aversive (footshock) | (-) SHA68 on NPS induced IA enhancement | (+) post-training (< 1 h) NPS injection effect on long-term (48–96h) consolidation of IA and retrieval in object memory; (/) pre-training effect of NPS injection on IA performance; (-) pre-retrieval NPS injection effect on IA performance. | See Table S4a,  (-) propranolol effect on NPS-induced memory enhancement |
| Pañeda et al., 2009 | Mouse, CRF1 knock-out (KO) mice and their WT littermates (C57BL/6J 129/SvJ background) | cocaine hydrochloride, 0.5 mg/kg, 15 μl, i.v.;  NPS, 0.1, 0.2, 0.45 nM, i.c.v.; antalarmin (CRF1 antagonist), 30 mg/kg; 0.01 ml/g, s.c. | approx. 123 M (total from reported groups), adult - age or weight not specified | operant conditioning, self-administered and reinstatement, appetitive (cocaine) | N/A | (+) NPS reinstated extinguished cocaine-seeking behavior in a dose-dependent manner; (/) cocaine reinstatement stimulant effects of NPS in CRF1 KO mice; (-) CRF1 antagonist antalarmin on the increase of active lever responding in the reinstatement model | (/) locomotor stimulant effects of NPS in CRF1 KO mice; (-) anxiety-like effects in CRF1 knock-out mice after NPS; (-) CRF1 antagonist antalarmin effect on the locomotor activating properties of NPS; (/) CRF1 antagonist antalarmin effect on NPS anxiolytic actions |
| Sartori et al, 2016 | Mouse,129S1/SvImJ (S1);  Rat, rHAB and rLAB | NPS (1nmol) - i.c.v; D-Cycloserine (DCS, 15 mg/kg, rat and 30mg/kg, mice) - i.p. | adult M; exact number, age or weight not specified | conditioning and extinction, Pavlovian, cued (auditory), aversive (footshock) | N/A | (+) NPS pre-extinction effect on extinction learning up to 10 days in extinction-deficient 129S1/SvImJ strain; (+) effect of NPS pre-extinction combined with partial NMDAR agonist DCS post-extinction in the extinction-impaired mice; (+) effect of NPS and DCS mix before extinction training on extinction consolidation up to 5 weeks in extinction-impaired mice | (+) DCS alone has similar extinction enhancement properties as NPS with similar temporal limitation |
| Schmoutz et al., 2012 | Rat, Wistar, WT | RTI-118 (NPSR1 antagonist), 5–30 mg/kg during conditioning / 1–20 mg/kg before reinstatement, i.p.;  SHA68 (NPSR1 antagonist), 0–50 mg/kg, i.p.; saline, 1 ml/kg, or yohimbine, 2.5 mg/kg, i.p.; cocaine, 0.25 mg/kg/infusion during conditioning / 15 mg/kg before reinstatement, i.v. | 54 M, 330 g | operant conditioning, self-administered and reinstatement, appetitive (food or cocaine) | (-) prior SHA68 injection (50 mg/kg i.p.) on cocaine- and food-seeking reinstatement; (-) RTI-118 (10–30 mg/kg i.p.) on cocaine- seeking; (-) RTI-118 (30 mg/kg) on food-seeking; (-) RTI-118 pretreatment on cue-induced cocaine self- administration reinstatement (dose-dependent) | N/A | (-) RTI-118 pretreatment on cocaine-associated lever pressing induced by 2.5 mg/kg yohimbine |
| Shao et al., 2016 | Mouse, C57BL/6J | scopolamine (muscarinic cholinergic receptor antagonist), 2mg/kg, i.p.; MK801, 0.1mg/kg, i.p.; NPS, 0.5nmol, i.c.v.; [D-Val5]NPS (NPSR1 antagonist), 40nmol, i.c.v. | 92 M, 6 wks | episodic memory, olfactory-spatial memory test, appetitive (novelty) | N/A | (+) NPS (0.5 nmol) rescued scopolamine- or MK801-impaired retrieval of odor-discrimination | (+) NPS on c-Fos expression in the subiculum complex, with ~ 90% of neurons being NPSR1-immunoreactive |
| Shirayama et al., 2015 | Rat, Sprague Dawley, WT | NPS, 10 or 1 pmol/side or SHA68 (NPSR1 antagonist), 100 or 10 pmol/side, i-NAc and -BNST | approx. 111 M (total from reported groups), 200–230 g | conditioning, learned helplessness (uncontrollable footshock stress), aversive (footshock) | (+) SHA68 i-BNST (100 pmol/side) on conditioned avoidance | (+) NPS (10 pmol/side) i-NAc shell on conditioned avoidance, reversed by NPSR1 antagonist; (/) NPS (10 pmol/side) i-NAc core on conditioned avoidance; (/) NPS (10 pmol/side) i-BNST on conditioned avoidance | (/) i-NAc shell NPS (10 pmol/side) and SHA68 (100 pmol/side) i-BNST on locomotion or anxiety-like behavior in the OFT and PA |
| Slattery et al., 2015 | rHAB, rLAB, and rNAB;  CD1 mice (mNAB; Munich breeding; *Npsr1* KO mice | NPS, 0.1 or 1 nmol, i.c.v; NPSR1-A (NPSR1 antagonist), D-Cys(tBu)5-NPS, 10 nmol - i.c.v; in rats, 45 min before anxiety tests or 20 min before fear extinction training; in mice, 25 min before behavioral tests | approx. total from reported groups:164 adult M rats of 280–350 g, and adult M 96 mice of 30–35 g | conditioning and extinction, Pavlovian, cued (auditory), aversive (footshock) | (/) NPSR1-A on fear extinction or fear expression in low anxiety rats and mice | (+) NPS (i.c.v) on anxiety predisposition in extinction learning and fear expression in both high anxiety mice and rats | (-) expression of *Npsr1*mRNA within the PVN and Amy in high anxiety rats and mice; (+) *Npsr1* polymorphisms in high anxiety rats and mice; (+) threefold expression in rHABs compared with rLABs in LC; (+) NPS-induced cAMP response in the high anxiety rats and mice; (+) *Npsr1*-A on anxiety level in low anxiety rats and mice (in LDB and EPM) |
| Thomasson et al, 2017 | Mouse, C57Bl/6J, WT | mouse NPS, 0.1 nmol, i.c.v. | 102 M, 10–12 wks | short-term memory, alternating task in T-maze, appetitive (spatial exploration) | N/A | (+) NPS on spatial STM acquisition by mitigating detrimental effects of 20h sleep deprivation | (+) NPS (i.c.v.) on cFos expression after spontaneous alternation in IL but not Amy, Hipp or PL and Cg cortex |
| Thorsell et al, 2013 | Rat, Wistar, WT | NCGC00185684 (NPSR1 antagonist), dissolved in 10% Solutol, 10% N,N-dimethylacetamide, and 80% 10 mm PBS, 1 ml/kg, i.p.; EtOH, 3.5 g/kg volume - oral; LiCl, 64 mg/kg, i.p. | approx. 165 M (total from reported groups), 300–350 g | operant conditioning, self-administered and cue- and stress-induced reinstatement, appetitive (alcohol and saccharin); conditioning, place aversion, aversive (LiCl) | NCGC: (-) operant alcohol self- administration; (/) reinstatement following extinction. | N/A | NCGC: (-) alcohol-induced ERK-phosphorylation in the rat central Amy; (-) motivation for alcohol reward measured as progressive ratio responding; (/) locomotor activity |
| Ubaldi et al., 2016 | Rat, Wistar, WT | SB334867 (non-peptide Ox1 antagonist), 5 µg, 0.5 µl, bilaterally i-VTA, i-LC, i-PVN), or i-BNST; NPS, 0.1, 0.25, 1.0 nmol, bilaterally in the i-LH;  Hcrt-1/Ox-A, 0.5, 1.5 nmol, 0.5 µl, bilaterally i-PVN or i-BNST; 10% alcohol, 0.2% saccharin solution, and mixed (for operant training), oral | approx. 77 M (total from reported groups), 175–225 g | operant conditioning, self-administered and reinstatement, appetitive (alcohol) | N/A | (+) i-LH NPS injection on discriminative cue-induced reinstatement of alcohol seeking | (-) alcohol reinstatement effects of NPS by the Hcrt-1/Ox-A antagonist microinjected-PVN or i-BNST, but not i-VTA or i-LC; (+) NPS containing axons in close apposition to hypothalamic Hcrt-1/Ox-A positive neurons, with a significant proportion expressing NPS receptors; (+)  retrograde tracing in either PVN or BNST, bilaterally labeled the somata of Hcrt-1/Ox-A neurons; (+) i-BNST or i-PVN Hcrt-1/Ox-A injection on alcohol seeking |
| Wang et al., 2020 | Mouse, C57Bl/6J, WT | [DVAl5]NPS (NPSR1 antagonist), 10 and 20 nmol, i.c.v.; SHA68 (NPSR1 antagonist), 10 μM, i.p. | 115 adult M, 6 wks | episodic memory, olfactory-spatial memory test, appetitive (novelty) | (-) SHA68 (50 mg/kg, i.p. pre-test) on retrieval of spatial olfactory memory; (-) [D-Val5]NPS (20 nmol, i.c.v. pre-test) on retrieval of spatial olfactory memory | N/A | (-) both antagonists on the number of c-Fos immunoreactivity in NPSR1-immunoreactive neurons in the AON, Pir, Sub, PrS and PaS |
| Zhao et al., 2010 | Rat, Sprague Dawley, WT | NPS, 0.5 nmol, i.c.v. | number not specified, M, 220–250 g | episodic and spatial memory, Morris water maze task, appetitive (escape) | N/A | (+) NPS on spatial learning and memory impairments caused by 72 h sleep deprivation | (+) NPS on alleviating sleep deprivation memory impairments and on expression of the phosphorylated form of CREB in the Hipp |
| Zoicas et al, 2016 | Mouse, CD1, WT | NPS, 1, 10, or 50 nmol/2 ml, or NPSR1-A (D-Cys((t)Bu)(5)-NPS, 10 nmol/2 ml, i.c.v. | approx. 136 M (total from reported groups), 10 wks, 30–35 g | conditioning, social and auditory-cued fear memory, aversive (footshock); social preference or avoidance following social defeat test, aversive (social aggressor) | (/) NPSR1-A effect on SFC-induced social fear | (+) extinction - reversed social fear conditioning of unfamiliar conspecifics; (-) (dose-dependent) social defeat-induced avoidance of known aggressive conspecifics; (-) (both doses) social avoidance; (/) cued fear acquisition; (/) overall cued fear extinction; (+) (low dose) within-session extinction; (-) (high dose) within-session extinction. | NPS: (-) general anxiety; (/) locomotor activity; NPSR1-A: (/) general anxiety and locomotor activity |

**Legend:** (+): positive effect; (-) negative effect; (/): no effect; AcbSh = nucleus accumbens shell; aCSF = artificial cerebrospinal fluid; Amy = amygdala; AON = anterior olfactory nucleus; BSA = bovine serum albumin; Cg = cingulate cortex; CORT = corticosterone; CREB = cAMP-response element binding protein; CRF1 = corticotropin-releasing factor receptor 1; DMSO = dimethyl sulfoxide; EtOH = ethanol; F = female; Glu = glutamatergic; IA = inhibitory avoidance; i-BLA = intra-BasoLateral Amygdala; i-BNST = intra bed nucleus of the stria terminalis; i-CeA = intra-central amygdala; i.c.v. = intracerebroventricular; i-DMH = intra-dorsomedial hypothalamus; i-EPN = endopiriform nucleus; IL = infralimbic cortex; (i-)LA = (intra-)lateral amygdala; (i-)LH = (intra-)lateral hypothalamus; i-NAc = intra-nucleus accumbens; i.p. = intraperitoneal; i-PeF = intra-perifornical area of the hypothalamus; i-PVN = intra-paraventricular nucleus (of the hypothalamus); i-PVT = intra-paraventricular nucleus of the thalamus; i.v. = intravenous; i-VTA = intra-ventral tegmental area; Hipp = hippocampus; LS = lateral septum; M = male; Med = medial; m/rHAB = high anxiety-related behavior mouse/rat; m/rLAB = low anxiety-related behavior mouse/rat; m/rNAB = normal anxiety-related behavior mouse/rat; NCGC00185684 = NCGC; mRNA = messenger ribonucleic acid; NMDAR = N-methyl-D-aspartate receptor; OxR1/Hcrt-r1 = hypocretin-1/orexin-1 receptor; PA = passive avoidance; PaS = parasubiculum; PBS = phosphate buffered saline; Pir = piriform cortex; PL = prelimbic cortex; PrS = presubiculum; s.c. = subcutaneous; STM = short-term memory; Sub = subiculum.
